# Nanobodies targeting conserved epitopes on the major outer membrane protein of *Campylobacter* as potential tools for control of *Campylobacter* colonization

**DOI:** 10.1186/s13567-017-0491-9

**Published:** 2017-12-08

**Authors:** Charlotte Vanmarsenille, Inés Díaz del Olmo, Jelle Elseviers, Gholamreza Hassanzadeh Ghassabeh, Kristof Moonens, Didier Vertommen, An Martel, Freddy Haesebrouck, Frank Pasmans, Jean-Pierre Hernalsteens, Henri De Greve

**Affiliations:** 1 Structural Molecular Microbiology, VIB, Pleinlaan 2, 1050 Brussels, Belgium; 2 Structural Biology Brussels, Vrije Universiteit Brussel, Pleinlaan 2, 1050 Brussels, Belgium; 3 Genetische Virologie, Vrije Universiteit Brussel, Pleinlaan 2, 1050 Brussels, Belgium; 4 Department of Pathology, Bacteriology and Avian Diseases, Faculty of Veterinary Medicine, Ghent University, Salisburylaan 133, 9820 Merelbeke, Belgium; 5 VIB Nanobody Service Facility, Vrije Universiteit Brussel, Pleinlaan 2, 1050 Brussels, Belgium; 6 Faculty of Medicine and de Duve Institute, Université Catholique de Louvain, Avenue Hippocrate 74, 1200 Brussels, Belgium

## Abstract

*Campylobacter* infections are among the most prevalent foodborne infections in humans, resulting in a massive disease burden worldwide. Broilers have been identified as the major source of campylobacteriosis and reducing *Campylobacter* loads in the broiler caeca has been proposed as an effective measure to decrease the number of infections in humans. Failure of current methods to control *Campylobacter* in broilers stresses the urgency to develop novel mitigation measures. We obtained six nanobodies with a broad specificity, that recognize strains belonging to the two most relevant species, *Campylobacter jejuni* and *Campylobacter coli*. The target of the nanobodies was identified as the major outer membrane protein, a porin that contributes to bacterial virulence and viability. Multimerization of the nanobodies led to agglutination of *C. jejuni* cells, which may affect colonization in the chicken gut. These *Campylobacter*-specific nanobodies may be useful to develop a strategy for preserving chickens from *Campylobacter* colonization.

**Electronic supplementary material:**

The online version of this article (10.1186/s13567-017-0491-9) contains supplementary material, which is available to authorized users.

## Introduction

Worldwide, *Campylobacter* is one of the most common causes of gastroenteritis [1, 2]. Less than 500 bacteria are required to establish infection, hence small amounts present in food or water can cause human infections [3]. The *Campylobacter* species mostly associated with infection, in both industrialized and developing countries, are *C. jejuni* and *C. coli* [4]. Typical clinical symptoms of *Campylobacter* infections in humans are abdominal cramps, diarrhoea and fever. Usually, *Campylobacter* enteritis is a self-limiting disease and antibiotic treatment is only necessary in persistent cases [5, 6]. However, infections with *C. jejuni* can lead to severe complications like the Guillain–Barré syndrome, a paralyzing neuropathological disease [7, 8]. The favoured environmental reservoir of *C.* *jejuni* is the intestinal tract of poultry, that is considered the natural host of *C. jejuni*. The elevated body temperature of chickens (i.e. 42 °C) corresponds to the optimal growth temperature of *C. jejuni*, which makes them an outstanding reservoir for the bacteria [9]. *Campylobacter* mainly colonizes the mucus layer of the intestinal tract and is abundantly present in the caecum. Up to 10^9^ CFU/g faecal content can be reached [10, 11]. At slaughter age, up to 80% of the flocks worldwide are contaminated with *Campylobacter* bacteria. Consumption and handling of contaminated poultry meat or carcasses are the most common causes of *Campylobacter* infections in humans [12–15].

Since poultry plays such an important role in transmission, a decrease of the colonization of poultry by *Campylobacter* will lead to a reduction of *Campylobacter*-related enteritis cases in humans [16]. Hygiene and biosafety play an essential role in the control of *Campylobacter* infections in poultry, but these methods alone are not sufficiently effective and must be complemented with novel control approaches [17]. Still, no effective control measures are available to prevent or reduce the prevalence of *C. jejuni* in poultry during primary production [18, 19]. The addition of antibiotics to animal feed is not acceptable, as this leads to an increasing number of resistant strains which has serious consequences for the treatment of humans [20, 21]. Therefore, a wide range of alternative approaches have been screened. An effective vaccine has not yet been developed and the use of fatty acids, bioactive plant additives or probiotics did not lead to the desired in vivo effect [22–24]. Other alternatives, like the use of bacteriophages and bacteriocins, are more promising, however, more research is required [24–26]. Maternal anti-*Campylobacter* antibodies protect young chicks during the first 2–3 weeks [27, 28]. Consequently, the use of maternal antibodies, isolated from eggs of immunized hens, has been assessed for the passive immunization of infected chickens. The results were promising for the use as therapeutic treatment [29, 30].

In this study, we describe the isolation and characterization of nanobodies (Nb) recognizing multiple *Campylobacter* strains. Nanobodies are the antigen-binding domains of heavy chain antibodies found in Camelidae [31]. They possess a number of advantages over antibodies, which makes their use attractive for diagnostic and therapeutic purposes. Nanobodies interact with their antigen with high affinity and specificity, they are highly stable and soluble and can be expressed in microbial expression systems [32, 33]. The nanobodies isolated in this study bind with the major outer membrane protein (MOMP). Outer membrane proteins (OMP) play a major role in adhesion and invasion during *Campylobacter* infections [34]. Therefore nanobodies targeting these proteins could be interesting tools for the development of a control strategy. One of the outer membrane proteins important for virulence of *Campylobacter* is the MOMP, encoded by the *porA*-gene. MOMP is a conserved trimeric β-barrel porin involved in adhesion [35]. The porin is also essential for viability of *Campylobacter*, as its deletion results in a lethal phenotype [36, 37]. It is required for structural organization and stabilization of the outer membrane and it makes the diffusion possible of compounds, like nutrients and antibiotics, across the membrane [38, 39].

## Materials and methods

### Bacterial strains and growth condition

The bacterial strains that were used in this study are indicated in Table 1. *C. jejuni* KC40 was used as a reference strain. *C. jejuni* was grown on Nutrient Broth Nr.2, solidified with 1.5% agar (NB2, CM0067; Thermo Fisher Scientific) under microaerobic conditions (Oxoid™ CampyGen™, Thermo Fisher Scientific) at 42 °C for 48 h. *Escherichia coli* strains were cultured on LB medium (Duchefa Biochemie), supplemented with the appropriate antibiotics if necessary.

**Table 1:** **Bacterial strains used in this study**

Species	Strain	Source
*C. jejuni*	KC40	Environment chicken^a^*^+^
	7P-6.12	Chicken^a+^
	10C-6.1	Chicken^a+^
	10KF-1.16	Chicken^a+^
	10KF-4.12	Chicken^a+^
	10VTDD-8	Chicken^a+^
	KC59.1	Chicken^a^*
	KC64.1	Chicken^a^*
	KC67.1	Chicken^a^*
	KC84.1	Chicken^a^*
	KC96.1	Chicken^a^*
	KC101	Environment chicken^a^*
	Cam12/0214	Human^b^
	Cam12/0231	Human^b^
	Cam12/0146	Human^b^
	Cam12/0152	Human^b^
	Cam12/0173	Human^b^
	Cam12/0197	Human^b^
	Cam12/0156	Human^b^
	Cam12/0190	Human^b^
	Cam12/0202	Human^b^
	Cam12/0222	Human^b^
	Cam12/0183	Human^b^
*C. coli*	52/P	Chicken^c^
	70/P	Chicken^c^
	K43/5	Chicken^c^
	KC7	Chicken^c^
	MB3361	Chicken^c^
*E. coli*	TG1	[68]
	DH5α	[69]
	WK6	[70]

* The *fla*-DGGE analysis is described in Najdenski et al. [51].

^+^The MLST results are described in Hermans et al. [30].

^a^Isolates obtained from a poultry farm or a slaughterhouse, provided by Dr. Marc Heyndrickx (Institute for Agricultural and Fisheries Research, Technology and Food Science Unit—Food Safety, Melle, Belgium).

^b^Clinical isolates obtained from faeces of infected patients, provided by Dr. D. Martiny (Microbiology Department, Iris-lab, Brussels, Belgium). MLST analysis showed that the isolates belong to different clonal complexes (CC-21, CC-464, CC-21, CC-206, CC-48, CC-45) (D. Martiny, personal communication).

^c^Isolates obtained from chickens, provided by Dr. Marc Heyndrickx (Institute for Agricultural and Fisheries Research, Technology and Food Science Unit—Food Safety, Melle, Belgium).

### Phage library construction and selection of anti-*Campylobacter* nanobodies

A detailed protocol for immunization, generation of a nanobody library and selection by phage panning has been described [40]. *C. jejuni* KC40 cells were heat-inactivated at 55 °C for 1 h. An alpaca was injected six times with 1.6 × 10^8^ inactivated *C. jejuni* KC40. Peripheral lymphocytes were isolated from the blood of the immunized alpaca, from which RNA was isolated and converted to cDNA [40]. PCR on the cDNA was used to amplify the sequences encoding the variable domains of heavy chain antibodies. The resulting PCR fragments were cloned in the phage display vector pHen4 [41] and transformed in *E. coli* TG1 cells. Phage display was used for the selection of *Campylobacter*-specific nanobodies from the immune library. The phage library was panned twice against an outer membrane extract. Binding phages from the second panning were eluted and used for infection of *E. coli* TG1. From 95 individual *E. coli* TG1 transformants, a periplasmic extract was prepared and used in an ELISA, to confirm their specificity for the outer membrane extract of *Campylobacter*. Subsequently, the nanobody-encoding genes from 21 positive clones were amplified from the pHen4 plasmid for sequencing.

### Tagging of the nanobodies

The nanobodies were cloned in the pHen6C vector [42], designed for the introduction of a histidine-tag (His-tag) at the C-terminus. PCR was performed using In-Fusion primers IF-NB1 (5′-TGGCCCAGGTGCAGCTGCAGGAGTCTGGAG-3′) and IF-NB2 (5′-TGAGGAGACGGTGACCTGGGTCC-3′). For the introduction of the PCR fragments in the pHen6C, the vector was digested with *Pst*I and *Bst*EII and the cloning was carried out with the In-Fusion^®^ HD Cloning Kit (Takara Bio USA, Inc).

For the construction of the strep-tagged nanobodies, the pHen6C derivatives encoding the His-tagged nanobodies were used. The His-tag was removed using the *Bst*EII and *Eco*RI restriction enzymes. The strep-tag was introduced by ligation of annealed oligo’s (5′-GTCACCGTCTCCTCATGGAGCCACCCGCAGTTCGAAAAATAAGTTTAAACTAC-3′ and 5′-AATTTAGTTTAAACTTATTTTTCGAACTGCGGGTGGCTCCATGAGGAGAC G-3′) in the linearized pHen6C vectors.

The resulting constructs were transformed into CaCl_2_-competent *E. coli* DH5α [43] and transformants were selected on LB-agar plates supplemented with 100 µg/mL carbenicillin. Colonies were screened by PCR with the primers FP24 (5′-CGCCAGGGTTTTCCCAGTCACGAC-3′) and RP24 (5′–AGCGGATAACAATTTCACACAGGA-3′). PCR-positive colonies were sequenced to confirm that the constructs were correct.

### Expression and purification of anti-*Campylobacter* nanobodies

Clones encoding C-terminally His-tagged anti-*Campylobacter* nanobodies were introduced in *E. coli* WK6 for expression and purification. The bacterial cells were grown at 37 °C in LB medium supplemented with carbenicillin (100 µg/mL). When OD_660 nm_ 0.6–0.8 was reached, nanobody expression was induced at 30 °C by adding 1 mM isopropyl β-d-1 thiogalactopyranoside (Thermo Fisher Scientific). After overnight incubation, the periplasmic content was extracted and the nanobodies were purified.

His-tagged nanobodies were purified by nickel-affinity chromatography using HisTrap HP columns (GE Healthcare Life Sciences). The columns were equilibrated and washed with 20 mM Tris–HCl, 1 M NaCl, pH 8.0. Nanobodies were eluted using a linear gradient to 1 M imidazole. The eluted protein fractions were analysed by SDS-PAGE, using 12.5% acrylamide gels stained with Coomassie blue dye. The PageRuler™ Prestained Protein Ladder (Thermo Fisher Scientific) is used as a molecular weight marker. Fractions containing pure nanobodies were pooled and dialyzed against phosphate-buffered saline (PBS).

Strep-tagged nanobodies were purified using StrepTrap HP columns (GE Healthcare Life Sciences). These were equilibrated and washed with 100 mM Tris–HCl, 150 mM NaCl, 1 mM EDTA, pH 8.0 and nanobodies were eluted with a linear gradient to 2.5 mM desthiobiotin. Fractions containing pure nanobodies were dialyzed against PBS.

### Whole-bacterial cell ELISA

The interaction of the purified nanobodies, with different *C. jejuni* and *C. coli* isolates was assessed by means of a whole-cell ELISA. The bacteria were grown on NB2-agar plates and after 48 h the cells were harvested from the plates with PBS. The bacterial cells were centrifuged at 3600 *g* for 15 min and washed in PBS. Bacterial cells were fixed with 2.5% (v/v, final concentration) of methanol-stabilized 37% formaldehyde solution (Merck), followed by incubation for 100 min at 42 °C. Afterwards, the fixed bacterial cells were pelleted by centrifugation. The pellet was resuspended in coating buffer (150 mM Na_2_CO_3_, 46 mM NaHCO_3_) and the OD_660_ was adjusted to 0.3. From the suspension, 100 µL per well was used to coat a 96-well plate. After overnight incubation at 4 °C, the plates were washed five times with PBS + 0.05% Tween^®^-20. To reduce nonspecific interactions, 200 µL 5% (w/v) bovine serum albumin (BSA) was used per well and incubated for 2 h. Subsequently, the wells were washed five times and 100 µL/well of the anti-*Campylobacter* nanobodies (50 µg/mL) was added, followed by 1 h incubation at room temperature. Afterwards, mouse anti-Histidine tag monoclonal antibody (1:1000) (AbD Serotec) and goat anti-mouse IgG conjugated to alkaline phosphatase (AP) (1:5000) (Sigma-Aldrich) were added consecutively. The ELISA was developed by addition of 2 mg/mL para-nitrophenyl phosphate (p-NPP) in ELISA buffer (100 mM Tris–HCl, pH 9.5, 5 mM MgCl_2_, 100 mM NaCl). The OD was read at 405 nm.

### Preparation of outer membrane extract

*Campylobacter jejuni* KC40 was cultured on NB2-agar plates and the bacterial cells were resuspended in 10 mM HEPES pH 7.4. The cells were lysed by passing the culture twice through the Cell Cracker (Glen Creston, Stanmore, VK, UK) at 1000 psi (pound-force per square inch) at 4 °C. To remove cell debris, the disrupted cell suspension was centrifuged at 12 000 *g* at 4 °C for 10 min. The further isolation of the outer membranes in the supernatant was performed as described [44], with the exception that, after the sarkosyl treatment, the membranes were centrifuged for 30 min instead of 1 h. The obtained OMPs were stored in 20 mM HEPES pH 7.4 at −20 °C.

### Purification of the MOMP

The MOMP was purified from a total membrane extract. The outer membrane proteins were incubated in the presence of 0.3% n–octylpolyoxyethylene (octyl-POE) for 20 min at 4 °C. The insoluble proteins were pelleted by centrifugation at 100 000 *g* for 1 h. This step was repeated once, followed by two successive extraction steps with 0.5% octyl-POE. The obtained fractions were further purified using anion-exchange chromatography. A Resource Q column (GE Healthcare Life Sciences) was equilibrated with 20 mM sodium phosphate buffer pH 6.0 supplemented with 0.6% octyl-POE and the extracted proteins were loaded on the equilibrated column. Elution was achieved using a linear gradient to 1 M NaCl. The eluted fractions were analysed by SDS-PAGE, stained with Coomassie blue dye. Pure fractions were pooled and dialysed against 20 mM sodium phosphate buffer pH 7.6 supplemented with 0.6% octyl-POE.

### Interaction of nanobodies and outer membrane proteins in ELISA

The interaction of the nanobodies with the outer membrane extract and the purified MOMP was investigated using ELISA. Native or denatured outer membrane extract and purified MOMP were coated in a 96-well plate at a concentration of 1 µg/mL. The ELISA was further carried out as described above. For the ELISA with outer membrane extract, tenfold serial dilutions were made of the anti-*Campylobacter* nanobodies, starting from 50 µg/mL.

### Pull-down assay for antigen determination

To isolate OMP-Nb complexes, the Dynabeads^®^ His-Tag Isolation and Pulldown kit (Thermo Fisher Scientific) was used. Twenty microliter of nanobodies (1.0 mg/mL) was added to 500 µL of the isolated outer membrane proteins (1.2 mg/mL) and the mixture was incubated for 2 h at 4 °C on a roller. The His-tagged nanobodies interact with the Co^2+^-coated magnetic beads. The procedure was followed as described in the manual, with the exception that the samples containing the OMP-Nb complexes where incubated overnight with the Dynabeads at 4 °C. An SDS-PAGE was performed on the eluted complexes and the gel was stained with the SilverQuest™ Silver Staining Kit (Thermo Fisher Scientific). The relevant protein bands were excised from the gel, destained and digested with trypsin for further analysis by liquid chromatography–tandem mass spectrometry (LC–MS/MS) as described [45].

Western blotting was performed on denatured and non-denatured proteins. The non-denatured samples, were run on a 12.5% acrylamide gel in running buffer composed of 14.4 g glycine, 3.03 g Tris and 0.250 g SDS per litre. After electrophoresis, the proteins were either transferred to a polyvinylidene difluoride (PVDF) membrane, activated with methanol, or stained with Coomassie blue dye. After transfer, the membrane was washed five times with PBS + 0.2% Triton X-100, followed by an incubation of 1 h at 4 °C with blocking buffer (PBS + 10% milk powder). The washing step was repeated in between every incubation step. The washed PVDF membrane was then incubated with 5 µg/mL anti-*Campylobacter* nanobody, diluted in PBS + 0.2% Triton X-100 + 5% milk powder for 1 h at 4 °C. For the detection of the His-tagged nanobodies, a mouse anti-Histidine tag monoclonal antibody (1:1000) (AbD Serotec) was added and incubated for 1 h at 4 °C. The western blot was developed with a goat anti-mouse IgG conjugated to alkaline phosphatase (AP) (1:5000) (Sigma-Aldrich). After 1 h at 4 °C, substrate (50 µL NBT/BCIP in 100 mM Tris–HCl, pH 9.5, 5 mM MgCl_2_, 100 mM NaCl) was added to the membrane and incubated at 37 °C.

### Microscale thermophoresis (MST)

MST, an immobilization-free method, was used for the characterization of the Nb-MOMP interaction. This technique allows the study of interactions of biomolecules in solution. A NanoTemper Monolith NT.115 instrument (NanoTemper Technologies) was used for the binding experiments, wherein a temperature gradient is formed in glass capillaries by means of an infrared laser. The movement of the biomolecules along this gradient is affected by changes in size or charge and modifications in their hydration shell. For the saturation binding experiment, an anti-*Campylobacter* nanobody was fluorescently labelled via free amino-groups with Dylight 650 NHS Ester (Thermo Fisher Scientific). The labelled nanobody (constant concentration of 32 nM) was mixed with twofold serial dilutions of MOMP, ranging from 0.3 nM to 5.0 µM. The interaction of the proteins was measured in 20 mM sodium phosphate buffer pH 7.6 supplemented with 150 mM NaCl and 0.6% octyl-POE. The samples were loaded into the glass capillaries (Monolith™ NT.Automated Standard Treated Capillary Chips), the thermophoresis measurements were performed (MST power 10% and LED 100%) and the data were analysed using the NT Analysis software (NanoTemper Technologies). Data were normalized to ΔFnorm [‰] [46]. A competition experiment was used to determine whether an unlabelled nanobody can impede the interaction of the labelled nanobody and the MOMP. In this experiment, the unlabelled nanobody was used at a constant concentration of 10 µM and mixed with a twofold serial dilution of MOMP (0.3 nM to 5.0 µM). Afterwards, the labelled nanobody was added to the suspension at a constant concentration of 32 nM. The experiment was further carried out as described above.

### Saturation and competition binding assay

A saturation binding assay was performed using ELISA, to determine the interaction between a constant concentration MOMP (1 µg/mL) and tenfold serial dilutions of His-tagged Nb84, ranging from 5 × 10^−7^ to 5 × 10^1^ µg/mL. In a second experiment, the influence of competitors on this interaction was examined. Therefore MOMP (1 µg/mL) was coated in a 96-well plate, after which serial dilutions (from 5 × 10^−7^ to 5 × 10^1^ µg/mL) of strep-tagged anti-*Campylobacter* nanobodies, used as competitors, and a constant concentration of His-tagged Nb84 (5 × 10^−2^ µg/mL) were simultaneously added. Bound His-tagged nanobody was detected with a mouse anti-Histidine tag monoclonal antibody and goat anti-mouse IgG conjugated to alkaline phosphatase (AP).

### Sequence analysis of *porA*-gene of *C. jejuni* and *C. coli* isolates

To analyse the genetic variability of MOMP between different *Campylobacter* strains, the *porA*-gene was amplified with primers F3 (5′-ATGAAACTAGTTAAACTTAGTTTA-3′) and R3 (5′-GAATTTGTAAAGAGCTTGAAG-3′) [47]. A single colony of every isolate was resuspended in 50 µL deionized H_2_O and the suspension was frozen at −80 °C and subsequently boiled at 95 °C for 5 min. Of the suspension 1 µL was used as a template.

### Immunofluorescence microscopy

*Campylobacter jejuni* KC40 cells were fixed during 10 min with a final concentration (v/v) of 1.2% formaldehyde (methanol-stabilized, Merck). Fixed cells were spotted on 0.1% poly-l-lysine treated glass slides and dried. The microscope slides were treated with 5% BSA for 15 min, followed by the addition of 30 µL anti-*Campylobacter* nanobodies (50 µg/mL) and incubation for 1 h at room temperature. The bound nanobodies were detected in two consecutive steps. First, mouse anti-His monoclonal antibodies (1:200) (AbD Serotec) were added and incubated for 1 h at room temperature. Anti-mouse IgG conjugated to Alexa Fluor 488 (1:250) (Thermo Fisher Scientific) was then added for fluorescent labelling. After 1 h, the slides were washed with PBS and dried. As a negative control, anti-F4 nanobodies [48, 49] were used. In between every step, the glass plates were washed with PBS and dried.

### Multimerization of nanobodies

Selected nanobodies were coupled to Co^2+^-coated magnetic Dynabeads (Dynabeads^®^ His-Tag Isolation and Pulldown, Thermo Fisher Scientific) to make them multivalent, using the buffers described in the manual. Nanobodies (24 µg) were added to 300 µg of Dynabeads and the mixture was incubated at room temperature during 10 min on a roller. Hereafter, the beads were washed three times to remove the unbound nanobodies and the nanobody-coupled beads were stored in pull-down buffer at 4 °C.

*Campylobacter jejuni* KC40 cells were harvested from NB2-agar plates with NB2 medium and a suspension with OD_660_ 2.0 was used for the agglutination assay. The bacterial cells and nanobody-coupled beads were mixed in a 1:4 ratio in a final volume of 10 µL on a slide. The slides were incubated at room temperature and examined visually and by phase contrast microscopy for agglutination. As a negative control, the agglutination of *Campylobacter* bacteria in the presence of beads coupled with anti-F4 nanobodies was assessed [48, 49].

## Results

### Isolation of anti-*Campylobacter* nanobodies with a broad specificity

**Figure 1:**
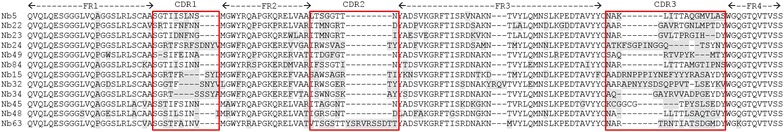
**Amino acid sequence alignment of anti-** ***Campylobacter*** **nanobodies selected for their broad specificity.** The structural framework regions are indicated by FR1–FR4 and the red boxes specify the CDRs. On the basis of the variation of the amino acid sequence of the CDR3, the nanobodies were divided in twelve unique groups.

To generate an immune library, an alpaca was immunized with heat-inactivated KC40 bacteria. From this library, 21 *Campylobacter*-specific nanobodies were obtained after two consecutive panning rounds against an outer membrane fraction of the *C. jejuni* strain KC40. These nanobodies belong to 12 different families (Figure 1) showing less than 80% identity in their complementarity-determining region 3 (CDR3), as defined by Pardon et al. [40]. A significant level of diversity is found among *Campylobacter* strains isolated from poultry [50]. For this reason, we selected one nanobody from each family and examined whether these display a broad detection range by testing their binding with *Campylobacter* strains [30, 51], belonging to different subtypes (Table 1). Whole-cell ELISA analysis showed that the six anti-*Campylobacter* nanobodies have a broad specificity range (Additional file 1). They interacted with all 23 tested *C. jejuni* strains, both from chickens and human patients, and with the 5 tested *C. coli* isolates. These data suggest that these nanobodies recognize conserved antigens on the cell surface of the *Campylobacter* strains.

### Nanobodies interact with folded outer membrane proteins

**Figure 2:**
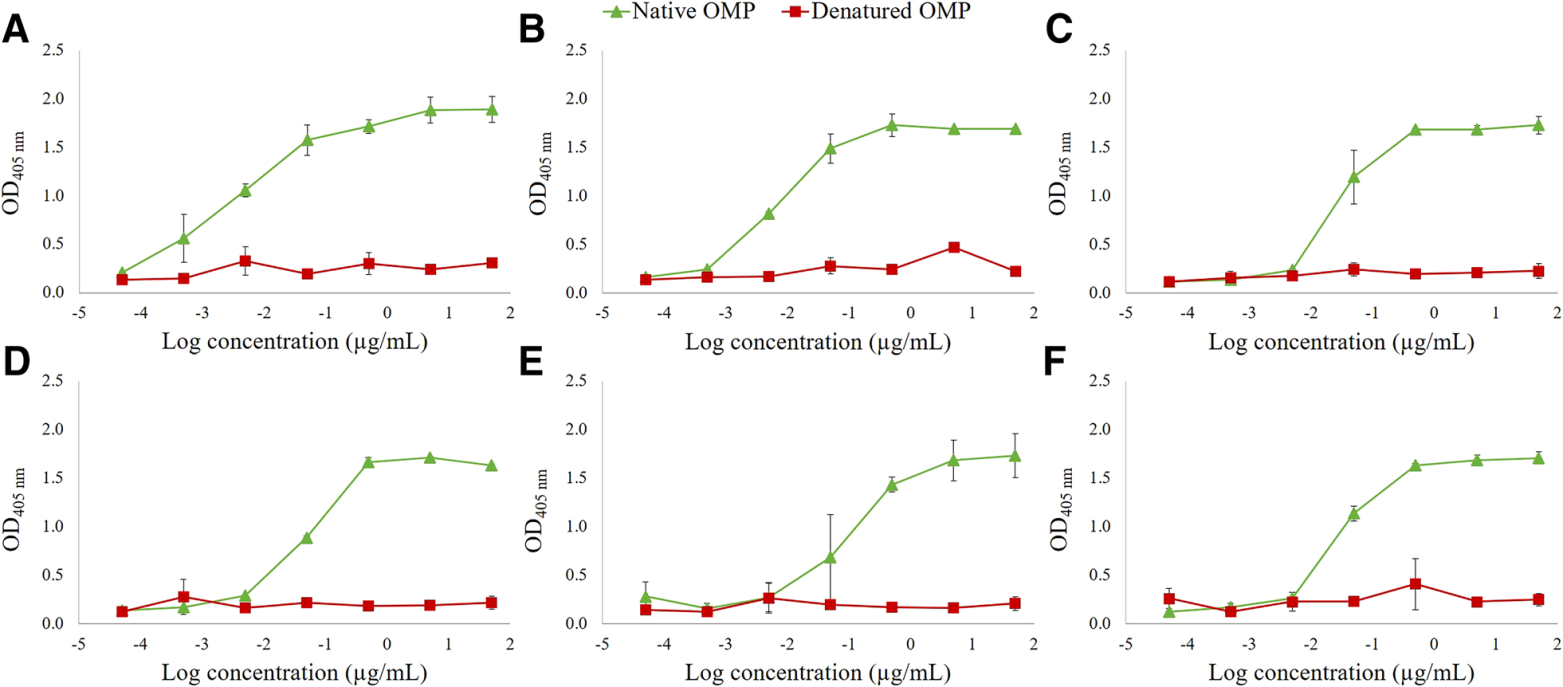
**Anti-** ***Campylobacter*** **nanobodies interact with native outer membrane proteins.** Serial tenfold dilutions of the nanobodies were used in ELISA to assess the binding with linear or conformational epitopes. OMPs (1 µg/mL) were coated in a 96-well plate and the interaction of His-tagged nanobodies with native, untreated OMP, and with denatured protein extract was measured. Binding of **A** Nb5, **B** Nb22, **C** Nb23, **D** Nb24, **E** Nb49 and **F** Nb84 was measured. For detection, mouse anti-Histidine tag monoclonal antibody and goat anti-mouse IgG conjugated to alkaline phosphatase were used. The error bars represent the standard deviations.

Whole-cell ELISA showed that the six nanobodies with broad specificity recognize epitopes exposed on the bacterial cell surface. ELISA was used to determine whether the nanobodies recognize conformational or linear epitopes (Figure 2). The nanobodies showed strong binding with native outer membrane proteins, while the interaction was significantly reduced after denaturation of the outer membrane proteins. These results indicate that the nanobodies bind to conformational epitopes present on outer membrane proteins.

### MOMP is the target of the isolated anti-*Campylobacter* nanobodies

**Figure 3:**
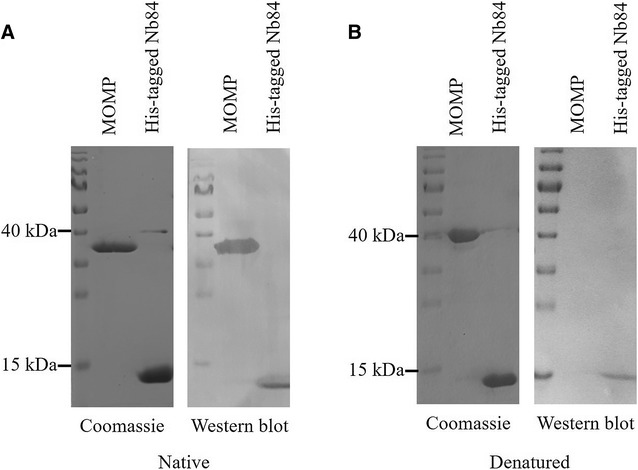
**Confirmation of the binding of Nb84 with native MOMP.** The purified MOMP monomer was subjected to non-denaturing (**A**) and denaturing (**B**) SDS-PAGE and transferred to a PVDF membrane for western blotting in which the membrane was first incubated with Nb84. Nb84 shows clear interaction with folded but not with unfolded MOMP. His-tagged Nb84 was added as a positive control. The interaction of the native protein with Nb84 was analysed using a mouse anti-Histidine tag monoclonal antibody and goat anti-mouse IgG conjugated to alkaline phosphatase. The PageRuler™ prestained protein ladder was used as a molecular weight marker.

To determine the antigens recognized by the six nanobodies with broad specificity, a pull-down experiment with magnetic beads was performed. Complexes with an outer membrane protein were isolated with three of the six nanobodies (Nb5, Nb22 and Nb84). The bound proteins were digested with trypsin and the peptides were analysed by LC–MS/MS. In each case, the amino acid sequences of the peptides corresponded to the MOMP sequence of *C. jejuni* NCTC 11168 (UniProtKB - P80672) (Additional file 2), a porin that is a crucial virulence factor of *Campylobacter*. The MOMP was subsequently purified from *C. jejuni* KC40 bacteria and binding with the three nanobodies was confirmed by western blotting. The binding of Nb84 with native MOMP is shown in Figure 3A. The western blot clearly shows a protein band corresponding to native MOMP. The western blot with the nanobodies shows no interaction with the denatured MOMP (Figure 3B). This confirms the interaction with surface-exposed epitopes of MOMP. Similar results were obtained for the five other nanobodies with broad specificity. ELISA further showed that the other nanobodies with less broad specificity also recognize native MOMP (Additional file 3).

### Affinity determination and competitive binding assay

**Figure 4:**
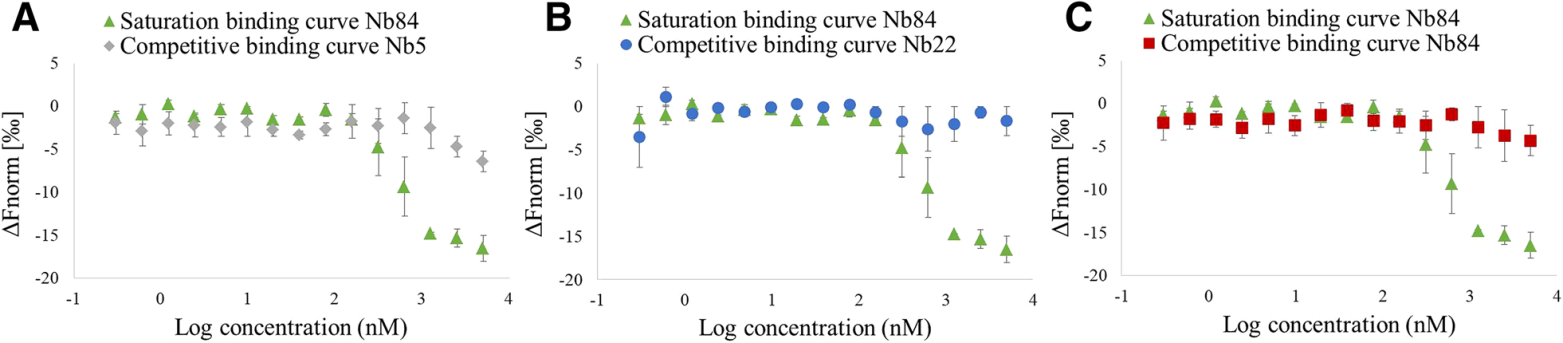
**MST analysis of the binding of anti-** ***Campylobacter*** **Nb84 with the purified MOMP monomer.** The binding curve of Nb84 with the MOMP monomer was obtained in a saturation binding experiment. The formation of Nb84-MOMP complexes was measured at constant concentrations of the fluorescently labelled Nb84 (32 nM) and varying concentrations of unlabelled MOMP (0.3 nM–5 µM). Data were normalized to ΔFnorm [‰]. The competitive binding curves visualise the inhibition of binding of the fluorescently labelled Nb84 (32 nM) with MOMP (0.3 nM–5 µM) by unlabelled **A** Nb5, **B** Nb22 and **C** Nb84 (10 µM). The error bars represent the standard deviations.

We determined the strength of the interaction between MOMP and the nanobodies Nb5, Nb22 and Nb84 using microscale thermophoresis (MST). Therefore, the nanobodies were fluorescently labelled and incubated with increasing concentrations of MOMP (Figure 4; Additional file 4). Changes in the thermophoretic properties upon complex formation were plotted, to obtain information on the dissociation constant (K_D_-value). Nb22 and Nb84 bind MOMP with intermediate nanomolar affinities (K_D_ = 118 ± 48 nM and K_D_ = 422 ± 159 nM, respectively). For Nb5, it was not possible to determine the dissociation constant because of a high signal to noise ratio.

The specificity of the binding curves was confirmed by adding an excess of the same unlabelled nanobody, which resulted in a loss of the MST signal (Figure 4C). In addition, a competitive MST assay was performed by adding an excess of a different unlabelled nanobody, that can potentially compete with the binding of the labelled nanobody to MOMP. When adding either unlabelled Nb5 or Nb22, no clear shift in the MST signal, corresponding to the binding of labelled Nb84 to MOMP, was observed (Figures 4A and B). These data show that these nanobodies do not bind the antigen simultaneously, suggesting that they may recognize the same, adjacent or overlapping epitopes. However, we cannot exclude the possibility that these nanobodies may still bind to different epitopes and interfere with each other’s binding by inducing conformational changes.

**Figure 5:**
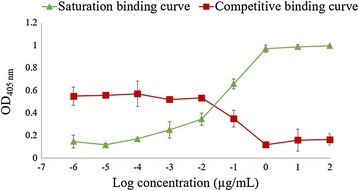
**Interaction between Nb84 and purified MOMP.** To obtain the saturation binding curve, ELISA plates were coated with purified MOMP monomers (1 µg/mL) and the interaction with increasing concentrations of His-tagged Nb84 (1 × 10^−6^ to 1 × 10^2^ µg/mL) was measured. A competition assay was performed to assess the inhibition of the interaction of His-tagged Nb84 with MOMP by increasing amounts of strep-tagged Nb84. His-tagged Nb84 (5 × 10^−2^ µg/mL) and a serial dilution of strep-tagged Nb84 (1 × 10^−6^ to 1 × 10^2^ µg/mL) were added to ELISA plates coated with MOMP (1 µg/mL). The ELISA was developed using a mouse anti-Histidine tag monoclonal antibody and goat anti-mouse IgG conjugated to alkaline phosphatase. The error bars represent the standard deviations.

To confirm the findings of the MST competition assay, we set up a competitive ELISA. The influence of strep-tagged nanobodies, of the 12 different families, on the binding of His-tagged Nb84 to MOMP was assessed (Figure 5; Additional file 5). Dose-dependent inhibition of the binding was confirmed for nine of the twelve nanobodies. For the other three nanobodies (Nb32, Nb34 and Nb45), it was shown that they could bind to MOMP simultaneously with Nb84, which indicates they recognize different epitopes.

### Variability in the *porA*-gene encoding MOMP

The MOMP of *C. jejuni* and *C. coli* is characterised by high genetic diversity. Little variability is observed in the transmembrane domains, in contrast to high variability in the extracellular loops [52]. The MOMP-encoding *porA*-gene of the 23 *C. jejuni* and the 5 *C. coli* stains was PCR-amplified and sequenced (Additional file 6). Based on the MOMP structure solved by Ferrara et al. [39], the extracellular loops and β-strands were identified in the sequence. Since the broad specificity range nanobodies bind to the surface of *Campylobacter* cells, we can assume that they interact with one of the surface-exposed loops of MOMP. These loops are however most variable amongst the sequenced MOMP genes. Sequence analysis shows that the extracellular loops L3 and L6 are the most conserved, in length and amino acid sequence, making these prime candidates to be recognized by the six nanobodies with a broad specificity range. Mapping of the sequences on the structure of MOMP confirms that the highest variability is located in the extracellular loops, while the sequence in the β-barrel structure is highly conserved (Additional file 7).

The sequence diversity in L3 and L6 in different *C. jejuni* and *C. coli* strains was analysed (Additional file 8). The MOMP encoding sequences in the MLSTdb database [53] were used for the analysis of the L6 region. Since the sequence of L3 is not present in the MLSTdb database, sequences were obtained from the NCBI database using Blastp with the *porA* sequence from *C. jejuni* KC40 as a query [54]. Comparison of the *porA* amino acid sequences, showed that the majority (81.9% or 1827 out of 2230) of analysed Loop 6 sequences and 92.5% (620 out of 670) of analysed Loop 3 sequences all carry amino acid substitutions at positions found in the L6 and L3 loops of the 28 MOMPs analysed in this study. From our study, we know that these amino acid substitutions in the L3 and L6 loops of MOMPs do not impede nanobody binding. The remaining 18.1% (403 of 2230) in case of L6 and 7.5% (50 of 670) encoding L3, contain amino acid changes on other positions. This does not imply that our six nanobodies with broad specificity would not bind these MOMP variants.

### Interaction of anti-*Campylobacter* nanobodies with the bacterial cell surface

**Figure 6:**
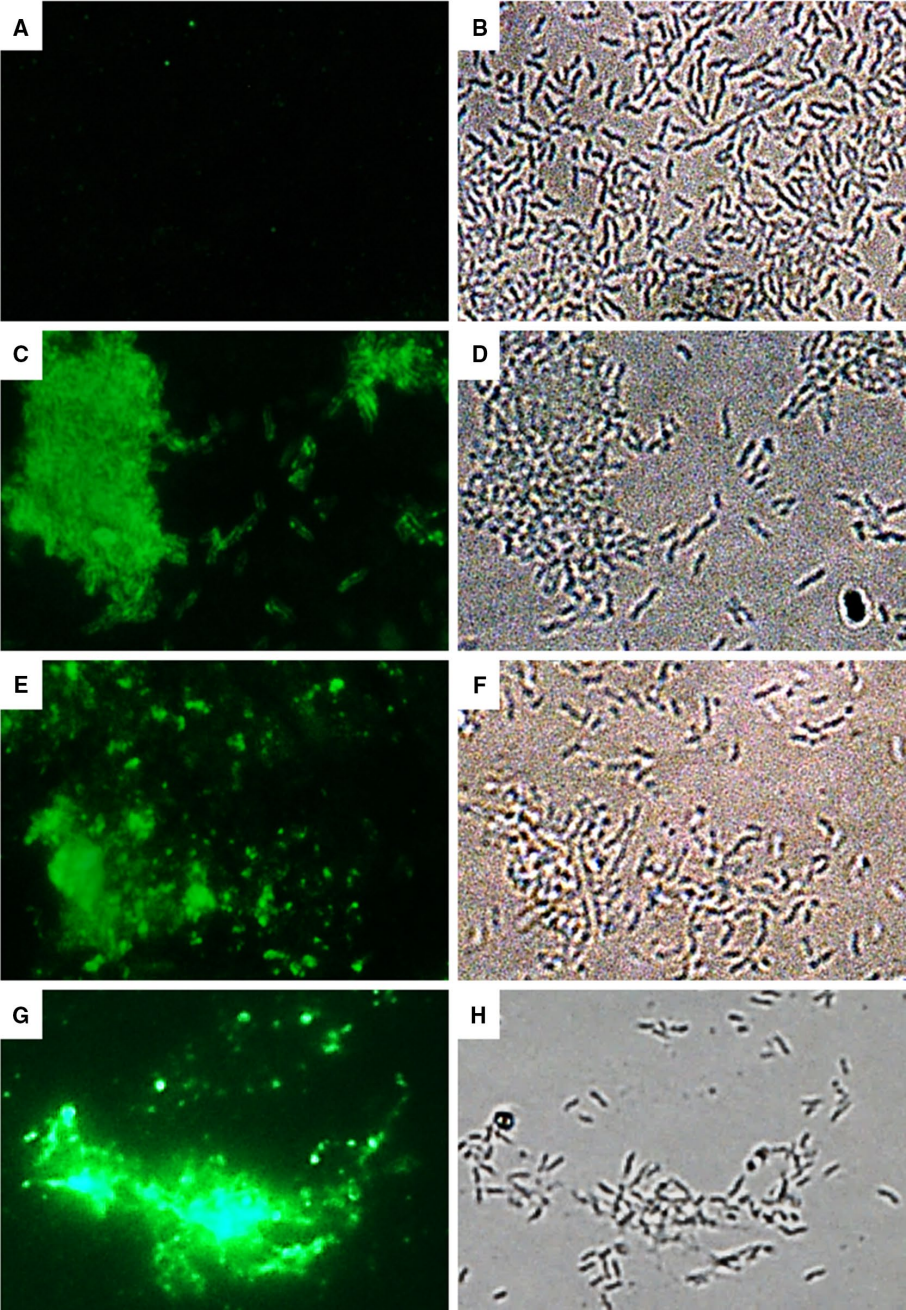
**Detection of the interaction of anti-** ***Campylobacter*** **nanobodies and** ***C. jejuni*** **KC40 by immunofluorescence microscopy.** The interaction was detected by (**A**, **C**, **E**, **G**) immunofluorescence microscopy and the *C. jejuni* cells (**B**, **D**, **F**, **H**) were visualised by bright field microscopy. **A**, **B** A nanobody specific for F4-fimbriated enterotoxigenic *E. coli* shows no binding with the *C. jejuni* cells. **C**, **D**; **E**, **F** and **G**, **H** The anti-*Campylobacter* nanobodies Nb22, Nb23 and Nb84 respectively, binds specifically with the *C. jejuni* cells.

Immunofluorescence microscopy was used to confirm the interaction of Nb22, Nb23 and Nb84 with the surface of *C. jejuni* KC40 cells (Figure 6). The results show clear fluorescence with the *Campylobacter*-specific nanobodies, in comparison with an anti-*E. coli* nanobody that was used as a negative control.

**Figure 7:**
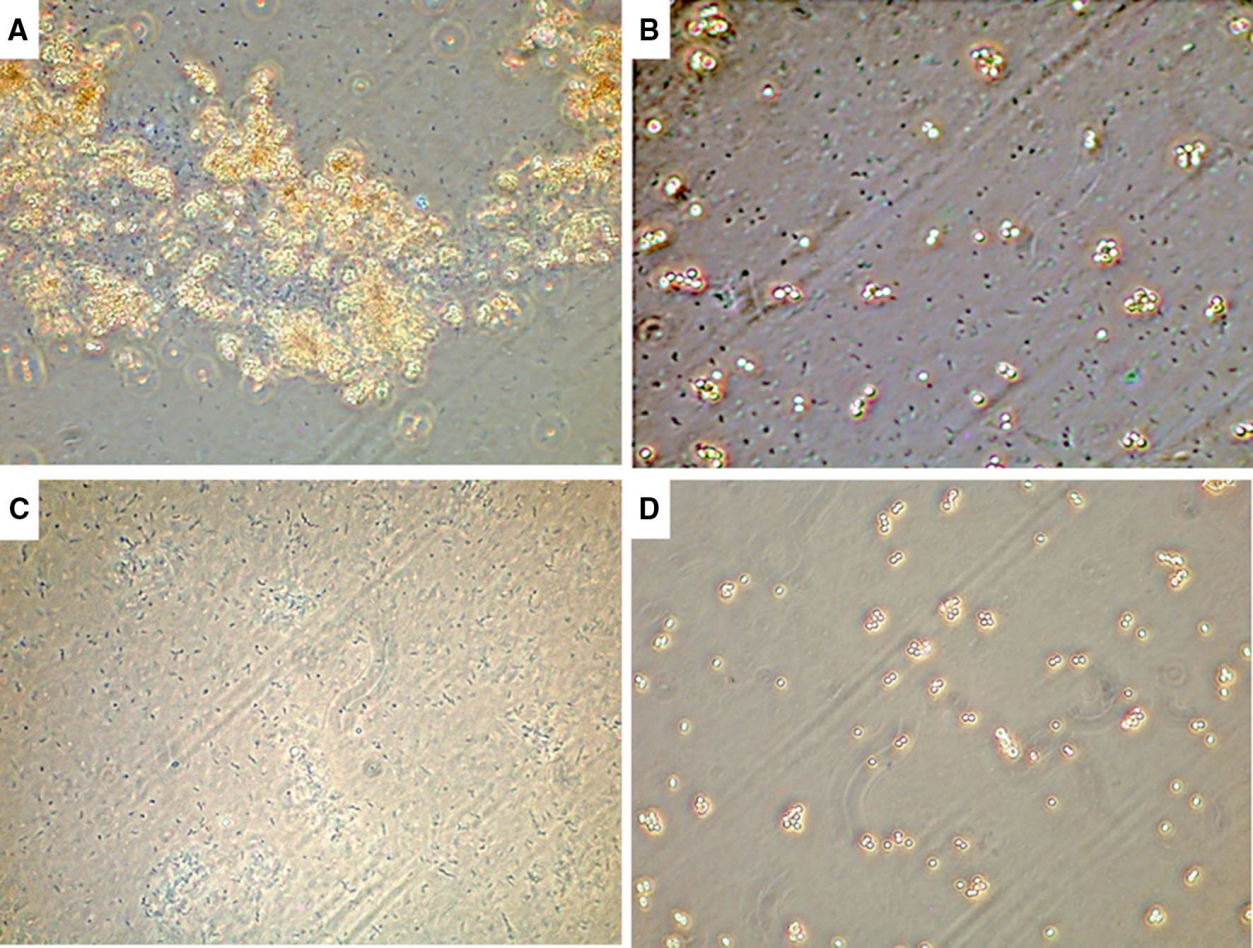
**Nanobody-coated beads agglutinate** ***C. jejuni*** **KC40 cells.** The His-tagged nanobodies were coupled to magnetic dynabeads, leading to multimerization. **A** Nb84 coupled to dynabeads causes agglutination of KC40 cells. **B** As a negative control, dynabeads coated with anti-*E. coli* nanobodies were mixed with KC40 cells. No agglutination was observed in this case. **C** The *C. jejuni* KC40 bacteria and **D** the beads coated with Nb84. The results were observed by phase contrast microscopy, using a ×100 oil immersion objective.

To explore whether the anti-*Campylobacter* nanobodies could agglutinate living *C. jejuni* KC40 cells, the six selected nanobodies were made multivalent, by coupling them to magnetic Dynabeads via their histidine tag. Clear agglutination was observed when *C. jejuni* KC40 cells were added (Figure 7A). Here the results of Nb84 with *C. jejuni* KC40 are shown. Beads, coated with an anti-*E. coli* nanobody, were used as a negative control and caused no visible agglutination of *Campylobacter* (Figure 7B). Additional negative controls were the *Campylobacter* bacteria and the beads coated with nanobodies alone (Figures 7C and D). The results confirm that the *Campylobacter*-specific nanobodies can bind with surface-exposed epitopes of *C. jejuni* KC40 and agglutinate the latter.

## Discussion

Substantial diversity of *Campylobacter* strains is found within broiler flocks and broilers are often colonized with multiple strains of *C. jejuni* [55]. Co-colonization by *C. jejuni* and *C. coli* has also been observed [13, 56]. Phage display technology was used to obtain a nanobody-library against the *C. jejuni* strain KC40. Here we selected six anti-*Campylobacter* nanobodies that interact with the cell surface of 23 different *C. jejuni* isolates derived from a poultry farm, poultry carcasses or faeces of human patients, as well as with 5 *C. coli* isolates.

Next, MOMP was identified as the antigen recognized by twelve nanobodies, from which six show a broad specificity. Since MOMP is an abundant protein in the outer membrane and since it is highly immunogenic [57], these findings are not surprising. MOMP is an essential porin for *Campylobacter* bacteria, as deletion of the *porA*-gene is lethal, and it is a key virulence factor, crucial for colonization [36, 37]. Islam et al. showed that the administration of recombinant MOMP, leads to protection of mice against colonization by heterologous *C. jejuni* strains [58]. Nanobodies targeting MOMP may interfere with its function and reduce colonization. Sequence analysis of the *porA*-gene of the 28 isolates showed that the extracellular Loop 3 and Loop 6 are the most conserved. These loops are potential candidates to be recognized by the nanobodies. The sequence diversity in these loops in the publicly available *porA*-sequences, was analysed. The results indicate that 92.5% of the analysed Loop 3 and 81.9% of the analysed Loop 6 sequences, carry amino acid substitutions at positions also found in the Loop 3 and Loop 6 sequences of the 28 isolates used in this study and do not influence the nanobody binding.

The anti-*Campylobacter* nanobodies were shown to interact with conformational epitopes on MOMP. Competition experiments showed that nine of the twelve anti-*Campylobacter* nanobodies could compete with each other’s binding to MOMP. This is an indication that they recognize the same or overlapping epitopes. Three of the anti-*Campylobacter* nanobodies were not able to inhibit the interaction of Nb84 with MOMP, hence they can simultaneously bind with MOMP. For Nb5, Nb22 and Nb84, affinity measurements were performed. Nb22 and Nb84 were shown to interact with MOMP with high-nanomolar affinities. However affinity measurements with Nb5 gave no consistent results. The binding strength of the nanobodies can presumably be further increased by the generation of a bivalent construct. By using a flexible peptide linker, dimers can be formed by coupling two nanobodies with the same specificity, resulting in higher avidity, or by combining two nanobodies binding different epitopes, creating bi-specific constructs [59].

The monovalent character and the small size of nanobodies, lead to rapid clearance. Multimerization of nanobodies can increase the retention. The results show that multimerization of the anti-*Campylobacter* nanobodies by non-covalent coupling to magnetic beads, resulted in agglutination of *C. jejuni* cells. Nanobodies can be multimerized by surface-expression on Generally Recognized As Safe (GRAS) organisms [60]. Agglutination typically takes place at high bacterial densities [61]. In the gut agglutination of *Campylobacter* could promote its removal and reduce colonization [62, 63]. Alternatively, Moor et al. showed that high-avidity IgA promotes the enchained growth of bacterial cells, leading to the formation of clumps. The latter mainly takes place at lower cell densities and leads to an enhanced clearance of the bacteria [61]. Virdi et al. [49] fused nanobodies against F4 fimbriae of enterotoxigenic *E. coli* (ETEC), with the Fc-domain of porcine IgA. *Arabidopsis thaliana* seeds were used for the expression of these constructs and passive vaccination with the seeds resulted in the protection of weaned piglets against ETEC-infections. Chicken feed supplemented with seeds containing anti-MOMP nanobodies, fused to the effector domains of chicken IgA or IgY, may, in a similar way, be used as a therapeutic agent against *Campylobacter* infection. The work described in this study, forms the basis for the development of such chimeric antibodies. The advantages of seeds include inexpensive production by standard agricultural practices, excellent stability of the antibodies in the seeds and the possible protection of the nanobodies against degradation in the gastrointestinal tract of the chickens [64]. Riazi et al. [65] reported that pentamers of flagella-specific nanobodies interfere with the motility of *C. jejuni* and lead to the reduction of colonization by *C. jejuni* in the caecum of treated chickens.

In conclusion, we isolated nanobodies against the essential virulence factor MOMP. Since these recognize conserved epitopes, present on *C. jejuni* and *C. coli* strains, they could potentially be used in therapy and as a diagnostic tool [49, 65–67].

## Additional files

**Additional file 1.** **Results of the ELISA performed to measure the binding of anti-** ***Campylobacter*** **nanobodies to different** ***Campylobacter*** **isolates.** Whole-cell ELISA was used to identify the anti-*Campylobacter* nanobodies with a broad specificity. *C. jejuni* isolates from poultry or faeces of human patients, as well as *C. coli* strains were tested.

**Additional file 2.** **LC–MS/MS identifies the MOMP as a target for the nanobodies.** Proteins recognized by the anti-*Campylobacter* nanobodies were isolated by a pull-down. After digestion of the proteins with trypsin, the peptides were analysed via LC-MS. The five peptides corresponding with the MOMP of *C. jejuni* NCTC 11168 (UniProtKB - P80672) are specified by the black boxes.

**Additional file 3.** **ELISA to determine the binding of anti-** ***Campylobacter*** **nanobodies to MOMP.** Purified MOMP (1 µg/mL) was coated and nanobodies were subsequently added at a concentration of 50 µg/mL. Mouse anti-Histidine tag monoclonal antibody and goat anti-mouse IgG conjugated to alkaline phosphatase were used for the development of the ELISA. The experiment was performed in duplicate and the mean of the obtained results is shown. The error bars represent the standard deviations. As a negative control, an anti-F4 nanobody was used.

**Additional file 4.** **Binding curve of Nb22 with purified MOMP obtained in a saturation binding experiment using MST analysis.** The formation of Nb22-MOMP complexes was measured at constant concentrations of the fluorescently labelled Nb22 (32 nM) and varying concentrations of unlabelled MOMP (0.3 nM to 5 µM). Data were normalized to ΔFnorm [‰]. The error bars represent the standard deviations.

**Additional file 5.** **ELISA to assess the interaction between** ***Campylobacter*** **-specific nanobodies and purified MOMP.** The saturation binding curve of the interaction between coated MOMP (1 µg/mL) and a His-tagged nanobody (1 × 10^−6^ to 1 × 10^2^ µg/mL) was obtained via ELISA. The dose-dependent inhibitory effect of a strep-tagged nanobody (1 × 10^−6^ to 1 × 10^2^ µg/mL) on the interaction between His-tagged Nb84 (5.10^−2^ µg/mL) and MOMP (1 µg/mL), is demonstrated in the competition binding curve. Inhibition by strep-tagged (A) Nb5, (B) Nb22, (C) Nb23, (D) Nb24, (E) Nb49, (F) 84, (G) Nb15, (H) Nb32, (I) Nb34, (J) Nb45, (K) Nb48 and (L) Nb63, was assessed. The ELISA was developed with mouse anti-Histidine tag monoclonal antibody and goat anti-mouse IgG conjugated to alkaline phosphatase. The error bars represent the standard deviations.

**Additional file 6.** **Alignment of amino acid sequences of the MOMP-encoding** ***porA*** **-gene.** The *porA* gene of the *C. jejuni* and *C. coli* isolates (Table 1) was amplified using PCR and aligned, using *C. jejuni* KC40 as a reference strain, to identify conserved regions. The PCR was performed with the primers F3 (5′-ATGAAACTAGTTAAACTTAGTTTA-3′) and R3 (5′-GAATTTGTAAAGAGCTTGAAG-3′). External loops are labelled from L1 to L7 and β-strands are underlined, based on the MOMP structure determined by Ferrara et al. [39]. Loops L3 and L6 are the most conserved in sequence and number of amino acids.

**Additional file 7.** **Sequence conservation of** ***porA*** **gene mapped on MOMP crystal structure.** The sequence conservation in the alignment of the *porA* gene of 28 *Campylobacter* isolates (Table 1), is visualised on the surface of MOMP. Blue corresponds to high amino acid sequence conservation and white with low conservation. High variability is observed in the extracellular loops, while the sequence encoding the transmembrane β-barrel is highly conserved. (Left) side view and (right) top view.

**Additional file 8.** **Sequence variability in L3 and L6 of the MOMP encoding sequences from** ***C. jejuni*** **and** ***C. coli*** **strains.** For the analysis of L3, sequences were obtained from the NCBI database using Blastp with the MOMP encoding sequence of *C. jejuni* KC40 as a query. The analysis of L6, was based on *porA* sequences in the MLSTdb database.

## Abbreviations

BSA: bovine serum albumin
CDR: complementarity-determining region
His: histidine
MST: microscale thermophoresis
Nb: nanobody
NB2: Nutrient Broth Nr.2
PBS: phosphate-buffered saline
PVDF: polyvinylidene difluoride
