# Supplementary material for: Nanobodies targeting conserved epitopes on the major outer membrane protein of Campylobacter as potential tools for control of Campylobacter colonization
Source: Vet Res. 2017 Dec 8;48:86. doi: 10.1186/s13567-017-0491-9 (PMC5721652; doi:10.1186/s13567-017-0491-9)
Supplement: Supplementary file 1 — Additional file 1. Results of the ELISA performed to measure the binding of anti- Campylobacter nanobodies to different Campylobacter isolates. Whole-cell ELISA was used to identify the anti-Campylobacter nanobodies with a broad specificity. C. jejuni isolates from poultry or faeces of human patients, as well as C. coli strains were tested. [file 13567_2017_491_MOESM1_ESM.pdf]

Results of the ELISA performed to measure the binding of anti-*Campylobacter* nanobodies to different *Campylobacter* isolates.

	Nb5	Nb22	Nb23	Nb24	Nb49	Nb84	Nb15	Nb32	Nb34	Nb45	Nb48	Nb63
KC40	++	++	++	++	++	++	++	+	++	++	++	++
7P-6.12	++	+	+	+	+	++	+	-	-	+	+	+
10C-6.1	++	+	+	++	+	++	+	-	-	+	+	+
10KF-1.16	++	++	++	++	++	++	++	+	+	++	++	++
10KF-4.12	++	+	+	+	+	++	-	-	-	-	+	+
10VTDD-8	++	+	+	++	+	++	+	-	-	+	+	+
KC59.1	++	++	++	++	++	++	++	++	+	++	++	++
KC64.1	++	+	+	++	+	++	+	-	-	+	+	+
KC67.2	++	+	+	+	+	++	+	-	-	+	+	+
KC84.2	+	+	+	++	+	++	+	-	-	+	+	+
KC96.1	++	++	++	++	+	++	+	+	-	++	+	+
KC101	++	++	++	++	++	++	+	+	+	++	+	+

	Nb5	Nb22	Nb23	Nb24	Nb49	Nb84
Cam12/0214	++	++	+	+	+	+
Cam12/0231	++	+	+	++	+	+
Cam12/0146	+	+	+	+	+	+
Cam12/0152	+	+	+	+	+	+
Cam12/0173	+	+	+	+	+	+
Cam12/0197	++	+	+	+	+	+
Cam12/0156	++	++	++	++	++	++
Cam12/0190	+	+	+	+	+	+
Cam12/0202	++	++	++	++	++	++
Cam12/0222	+	+	+	+	+	+
Cam12/0183	+	+	+	+	+	+

	Nb5	Nb22	Nb23	Nb24	Nb49	Nb84
52/P	++	+	++	+	+	+
70/P	++	++	++	++	++	++
K43/5	+	+	+	+	+	+
KC7	++	++	++	++	++	++
MB3361	+	+	+	+	+	+

(++) Values higher than two times the cut-off (Grey). (+) Values higher than the cut-off. (-) Values lower than the cut-off (Red). To calculate the cut-off, the following equation was used: cut-off = $\overline{x}_{neg} + (3\sigma_{neg})$. In this equation $\overline{x}_{neg}$ is the mean and σ_{neg} the standard deviation of the negative control. As a negative control, the ELISA was performed in the absence of anti-*Campylobacter* nanobodies.

Results of the ELISA performed to measure the binding of anti-*Campylobacter* nanobodies to different *Campylobacter* isolates.

	Nb5	Nb22	Nb23	Nb24	Nb49	Nb84	Nb15	Nb32	Nb34	Nb45	Nb48	Nb63
KC40	1.055 ± 0.039	0.980 ± 0.007	0.871 ± 0.050	0.932 ± 0.028	0.764 ± 0.226	0.948 ± 0.034	0.827 ± 0.054	0.280 ± 0.007	1.218 ± 0.215	0.969 ± 0.146	0.895 ± 0.094	0.664 ± 0.017
7P-6.12	0.675 ± 0.117	0.435 ± 0.012	0.390 ± 0.053	0.401 ± 0.044	0.438 ± 0.022	0.628 ± 0.035	0.402 ± 0.050	0.212 ± 0.012	0.239 ± 0.029	0.341 ± 0.031	0.398 ± 0.039	0.347 ± 0.007
10C-6.1	0.753 ± 0.044	0.455 ± 0.034	0.425 ± 0.007	0.549 ± 0.022	0.380 ± 0.034	0.651 ± 0.123	0.407 ± 0.046	0.222 ± 0.006	0.232 ± 0.024	0.424 ± 0.027	0.447 ± 0.005	0.372 ± 0.033
10KF-1.16	0.856 ± 0.038	0.736 ± 0.034	0.750 ± 0.016	0.755 ± 0.004	0.682 ± 0.082	0.912 ± 0.006	0.663 ± 0.020	0.362 ± 0.114	0.362 ± 0.029	0.704 ± 0.029	0.724 ± 0.019	0.584 ± 0.039
10KF-4.12	0.643 ± 0.109	0.427 ± 0.076	0.371 ± 0.069	0.445 ± 0.078	0.388 ± 0.138	0.674 ± 0.002	0.239 ± 0.052	0.217 ± 0.036	0.206 ± 0.007	0.191 ± 0.075	0.364 ± 0.040	0.339 ± 0.009
10VTDD-8	0.805 ± 0.047	0.458 ± 0.017	0.406 ± 0.121	0.618 ± 0.043	0.449 ± 0.022	0.846 ± 0.016	0.361 ± 0.182	0.231 ± 0.023	0.181 ± 0.037	0.440 ± 0.055	0.432 ± 0.033	0.473 ± 0.048
KC59.1	1.076 ± 0.142	1.049 ± 0.120	1.029 ± 0.092	1.019 ± 0.072	0.830 ± 0.175	1.165 ± 0.098	0.929 ± 0.051	0.835 ± 0.032	0.296 ± 0.097	0.976 ± 0.029	0.950 ± 0.001	0.744 ± 0.031
KC64.1	0.878 ± 0.133	0.470 ± 0.033	0.445 ± 0.058	0.605 ± 0.036	0.433 ± 0.037	0.729 ± 0.047	0.411 ± 0.012	0.234 ± 0.014	0.206 ± 0.004	0.437 ± 0.009	0.416 ± 0.016	0.371 ± 0.015
KC67.2	0.783 ± 0.112	0.471 ± 0.007	0.415 ± 0.059	0.399 ± 0.018	0.451 ± 0.087	0.735 ± 0.022	0.372 ± 0.007	0.243 ± 0.044	0.220 ± 0.015	0.440 ± 0.010	0.437 ± 0.038	0.374 ± 0.034
KC84.2	0.426 ± 0.100	0.456 ± 0.002	0.402 ± 0.001	0.621 ± 0.068	0.445 ± 0.017	0.620 ± 0.010	0.338 ± 0.002	0.230 ± 0.024	0.219 ± 0.010	0.428 ± 0.010	0.382 ± 0.029	0.376 ± 0.038
KC96.1	0.913 ± 0.065	0.725 ± 0.005	0.572 ± 0.014	0.717 ± 0.048	0.486 ± 0.003	0.757 ± 0.065	0.471 ± 0.055	0.342 ± 0.084	0.190 ± 0.052	0.557 ± 0.016	0.424 ± 0.043	0.440 ± 0.032
KC101	0.963 ± 0.053	0.797 ± 0.024	0.645 ± 0.008	0.668 ± 0.031	0.604 ± 0.013	0.769 ± 0.063	0.456 ± 0.046	0.393 ± 0.130	0.288 ± 0.013	0.483 ± 0.117	0.434 ± 0.041	0.451 ± 0.044

	Nb5	Nb22	Nb23	Nb24	Nb49	Nb84
Cam12/0214	0.435 ± 0.066	0.432 ± 0.080	0.369 ± 0.035	0.378 ± 0.044	0.358 ± 0.042	0.368 ± 0.025
Cam12/0231	0.478 ± 0.085	0.370 ± 0.078	0.361 ± 0.028	0.498 ± 0.086	0.356 ± 0.042	0.358 ± 0.026
Cam12/0146	0.339 ± 0.088	0.376 ± 0.118	0.324 ± 0.055	0.342 ± 0.050	0.356 ± 0.051	0.354 ± 0.049
Cam12/0152	0.401 ± 0.165	0.338 ± 0.072	0.301 ± 0.026	0.349 ± 0.044	0.355 ± 0.054	0.348 ± 0.050
Cam12/0173	0.325 ± 0.064	0.350 ± 0.044	0.389 ± 0.148	0.346 ± 0.036	0.358 ± 0.053	0.320 ± 0.043
Cam12/0197	0.434 ± 0.085	0.361 ± 0.027	0.371 ± 0.033	0.383 ± 0.032	0.364 ± 0.024	0.359 ± 0.037
Cam12/0156	0.448 ± 0.247	0.448 ± 0.231	0.486 ± 0.247	0.459 ± 0.282	0.490 ± 0.176	0.463 ± 0.288
Cam12/0190	0.342 ± 0.044	0.351 ± 0.091	0.330 ± 0.067	0.365 ± 0.067	0.329 ± 0.057	0.317 ± 0.052
Cam12/0202	0.831 ± 0.245	0.851 ± 0.306	0.823 ± 0.281	0.902 ± 0.297	0.762 ± 0.255	0.869 ± 0.330
Cam12/0222	0.359 ± 0.027	0.363 ± 0.047	0.357 ± 0.040	0.385 ± 0.022	0.355 ± 0.034	0.363 ± 0.021
Cam12/0183	0.345 ± 0.097	0.344 ± 0.084	0.362 ± 0.086	0.390 ± 0.075	0.352 ± 0.073	0.344 ± 0.098

	Nb5	Nb22	Nb23	Nb24	Nb49	Nb84
52/P	0.942 ± 0.041	0.401 ± 0.016	0.911 ± 0.033	0.440 ± 0.049	0.387 ± 0.048	0.386 ± 0.008
70/P	0.776 ± 0.089	0.717 ± 0.064	0.922 ± 0.165	0.838 ± 0.113	0.757 ± 0.136	0.800 ± 0.021
K43/5	0.324 ± 0.024	0.322 ± 0.029	0.276 ± 0.004	0.433 ± 0.058	0.294 ± 0.079	0.320 ± 0.036
KC7	2.057 ± 0.238	2.048 ± 0.131	2.063 ± 0.030	1.944 ± 0.036	2.388 ± 0.033	2.389 ± 0.014
MB3360	0.426 ± 0.059	0.423 ± 0.034	0.459 ± 0.045	0.426 ± 0.052	0.384 ± 0.013	0.420 ± 0.025

Mean ± standard deviation
