# Supplementary figures and images for: Nanobodies targeting conserved epitopes on the major outer membrane protein of Campylobacter as potential tools for control of Campylobacter colonization
Source: Vet Res. 2017 Dec 8;48:86. doi: 10.1186/s13567-017-0491-9 (PMC5721652; doi:10.1186/s13567-017-0491-9)

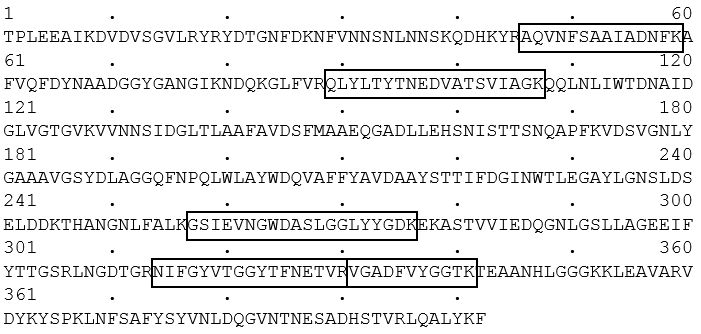

Supplement: Supplementary file 2 — Additional file 2. LC–MS/MS identifies the MOMP as a target for the nanobodies. Proteins recognized by the anti-Campylobacter nanobodies were isolated by a pull-down. After digestion of the proteins with trypsin, the peptides were analysed via LC-MS. The five peptides corresponding with the MOMP of C. jejuni NCTC 11168 (UniProtKB - P80672) are specified by the black boxes. [file 13567_2017_491_MOESM2_ESM.jpg]

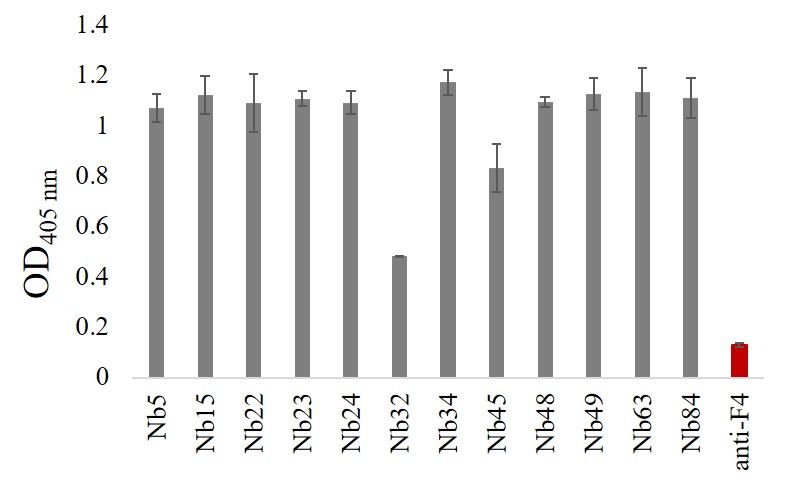

Supplement: Supplementary file 3 — Additional file 3. ELISA to determine the binding of anti- Campylobacter nanobodies to MOMP. Purified MOMP (1 µg/mL) was coated and nanobodies were subsequently added at a concentration of 50 µg/mL. Mouse anti-Histidine tag monoclonal antibody and goat anti-mouse IgG conjugated to alkaline phosphatase were used for the development of the ELISA. The experiment was performed in duplicate and the mean of the obtained results is shown. The error bars represent the standard deviations. As a negative control, an anti-F4 nanobody was used. [file 13567_2017_491_MOESM3_ESM.jpg]

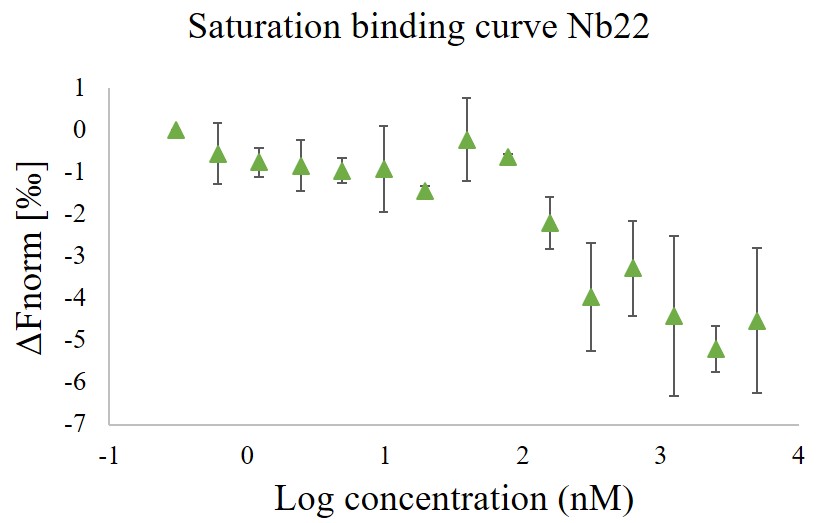

Supplement: Supplementary file 4 — Additional file 4. Binding curve of Nb22 with purified MOMP obtained in a saturation binding experiment using MST analysis. The formation of Nb22-MOMP complexes was measured at constant concentrations of the fluorescently labelled Nb22 (32 nM) and varying concentrations of unlabelled MOMP (0.3 nM to 5 µM). Data were normalized to ΔFnorm [‰]. The error bars represent the standard deviations. [file 13567_2017_491_MOESM4_ESM.jpg]

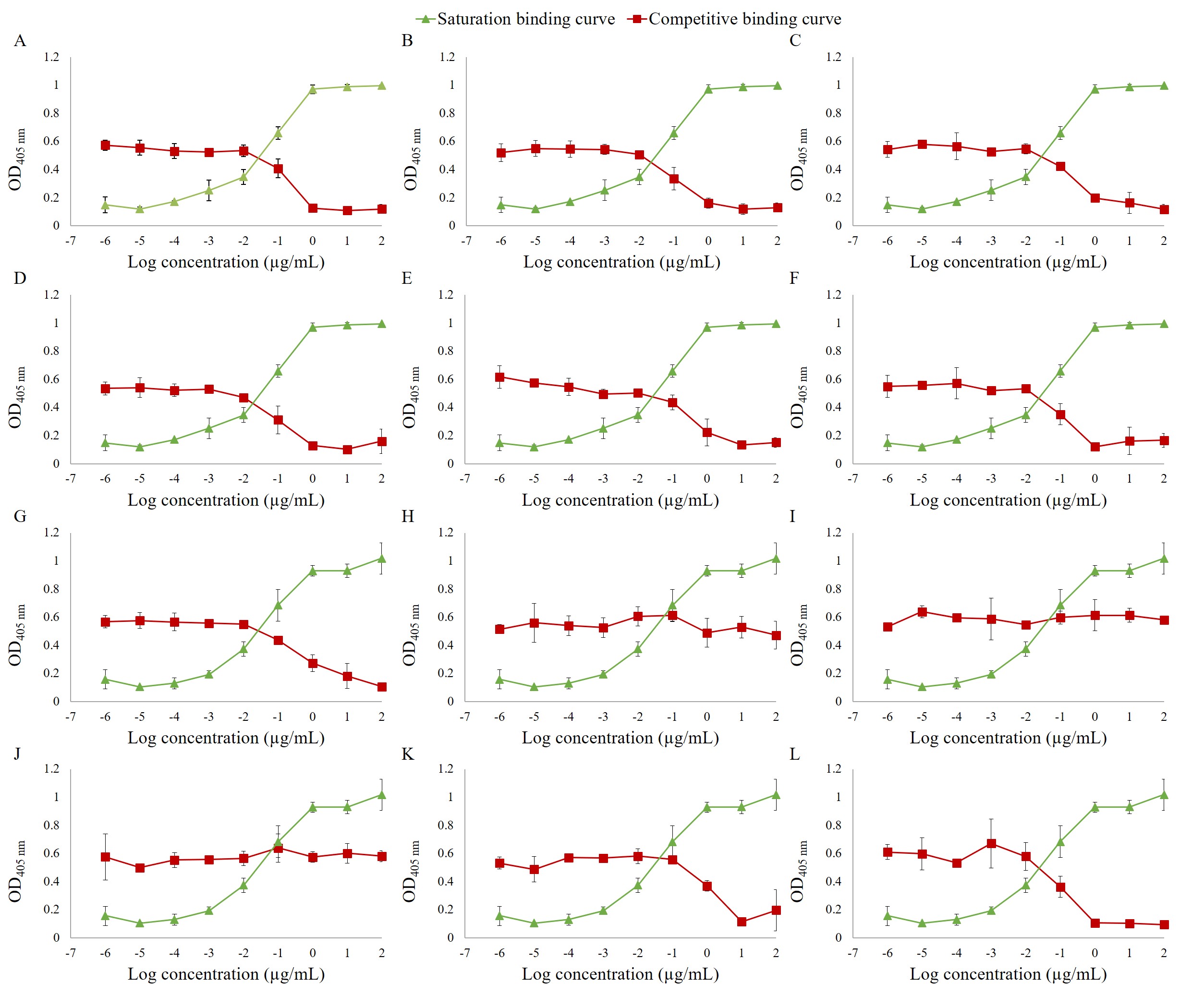

Supplement: Supplementary file 5 — Additional file 5. ELISA to assess the interaction between Campylobacter -specific nanobodies and purified MOMP. The saturation binding curve of the interaction between coated MOMP (1 µg/mL) and a His-tagged nanobody (1 × 10^−6^ to 1 × 102 µg/mL) was obtained via ELISA. The dose-dependent inhibitory effect of a strep-tagged nanobody (1 × 10^−6^ to 1 × 102 µg/mL) on the interaction between His-tagged Nb84 (5.10−2 µg/mL) and MOMP (1 µg/mL), is demonstrated in the competition binding curve. Inhibition by strep-tagged (A) Nb5, (B) Nb22, (C) Nb23, (D) Nb24, (E) Nb49, (F) 84, (G) Nb15, (H) Nb32, (I) Nb34, (J) Nb45, (K) Nb48 and (L) Nb63, was assessed. The ELISA was developed with mouse anti-Histidine tag monoclonal antibody and goat anti-mouse IgG conjugated to alkaline phosphatase. The error bars represent the standard deviations. [file 13567_2017_491_MOESM5_ESM.jpg]

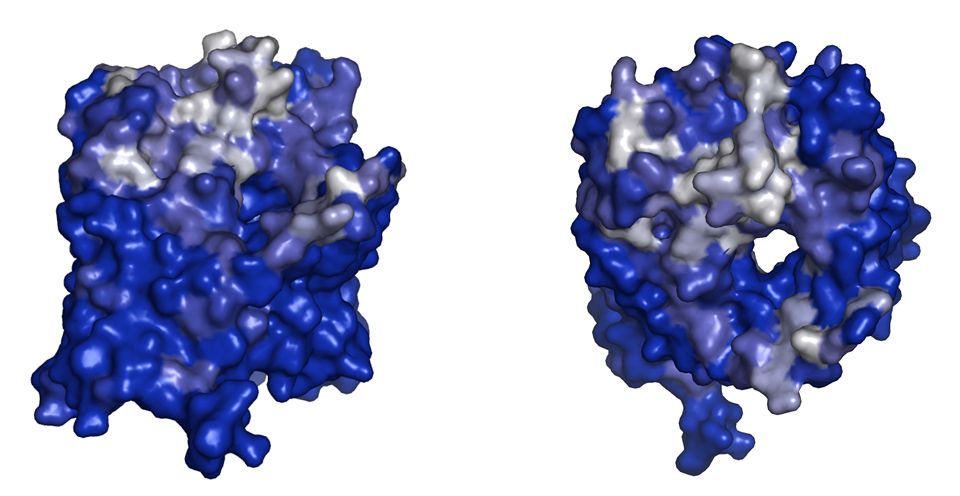

Supplement: Supplementary file 7 — Additional file 7. Sequence conservation of porA gene mapped on MOMP crystal structure. The sequence conservation in the alignment of the porA gene of 28 Campylobacter isolates (Table 1), is visualised on the surface of MOMP. Blue corresponds to high amino acid sequence conservation and white with low conservation. High variability is observed in the extracellular loops, while the sequence encoding the transmembrane β-barrel is highly conserved. (Left) side view and (right) top view. [file 13567_2017_491_MOESM7_ESM.jpg]
