# Supplementary material for: Nanobodies targeting conserved epitopes on the major outer membrane protein of Campylobacter as potential tools for control of Campylobacter colonization
Source: Vet Res. 2017 Dec 8;48:86. doi: 10.1186/s13567-017-0491-9 (PMC5721652; doi:10.1186/s13567-017-0491-9)
Supplement: Supplementary file 6 — Additional file 6. Alignment of amino acid sequences of the MOMP-encoding porA -gene. The porA gene of the C. jejuni and C. coli isolates (Table 1) was amplified using PCR and aligned, using C. jejuni KC40 as a reference strain, to identify conserved regions. The PCR was performed with the primers F3 (5′-ATGAAACTAGTTAAACTTAGTTTA-3′) and R3 (5′-GAATTTGTAAAGAGCTTGAAG-3′). External loops are labelled from L1 to L7 and β-strands are underlined, based on the MOMP structure determined by Ferrara et al. [39]. Loops L3 and L6 are the most conserved in sequence and number of amino acids. [file 13567_2017_491_MOESM6_ESM.pdf]

```
KC40       tpleeaikdvdvsgvlryrydtgn--fdknf-i--nnsnlnnskqdhkyraqvnfsaaiadnfkafvqfdynaadg
7P-6.12    tpleeaikdvdvsgvlryrydtgn--fdknf-v--nnsnlnnnkqdhnyraqvnfsaaiadnfkafiqfdynavdg
10C-6.1    tpleeaikdvdvsgvlryrydtgn--fdknf-v--nnsnlnnnkqdhkyraqvnfgaaiadnfkafiqfdynavdg
10KF-1.16  tpleeaikdvdvsgvlryrydtgn--fdknf-v--nnsnlnnskqdhkyraqvnfsaaiadnfkafvqfdynaadg
10KF-4.12  tpleeaikdidvsgvlryryessn--pwsna-n--fgsgi-sgkqdhkyraqvnfsgaisdnfkafvqfdynsqdg
10-VTDD8   tpleeaikdvdvsgvlryrydtgn--fdknf-v--nnsnlnnnkqdhnyraqvnfsaaiadnfkafiqfdynavdg
KC59.1     tpleeaikdvdvsgvlryrydtgn--fdknf-v--nnsnlnnskqdhkyraqvnfsaaiadnfkafvqfdynaadg
KC64.1     tpleeaikdvdvsgvlryryessn--pwsna-n--fgsgi-sgkqdhkyraqvnfsgaisdnfkafvqfdynsqdg
KC67.2     tpleeaikdvdvsgvlryrydtgn--fdknf-v--nnsnlnnskqdhkyraqvnfsaaiadnfkafvqfdynaadg
KC84.2     tpleeaikdidvsgvlryryessn--pwsna-n--fgsgi-sgkqdhkyraqvnfsgaisdnfkafvqfdynsqdg
KC96.1     tpleeaikdvdvsgvlryrydtgn--fdknf-v--nnsnlnnskqdhkyraqvnfsaaiadnfkafvqfdynaadg
KC101      tpleeaikdvdvsgvlryrydtgn--fdknf-v--nnsnlnnskqdhkyraqvnfsaaiadnfkafvqfdynaadg

Cam12/0214 tpleeaikdvdvsgvlryrydtgn--fdknf-v--nnsnlnnskqdhkyraqvnfsaaiadnfkafvqfdynaadg
Cam12/0231 tpleeaikdvdvsgvlryrydtgn--fdknf-v--nnsnlnnnkqdhkyraqvnfsaaiadnfkafiqfdynavdg
Cam12/0146 tpleeaikdvdvsgvlryrydtgn--fdknf-v--nnsnlnnskqdhkyraqvnfsaaiadnfkafvqfdynaadg
Cam12/0152 tpleeaikdvdvsgvlryrydtgn--fdknf-v--nnsnlnnnkqdhkyraqvnfsaaiadnfkafiqfdynavdg
Cam12/0173 tpleeaikdidvsgvlryryessn--pwsna-n--fgsgi-sgkqdhkyraqvnfsgaisdnfkafvqfdynsqdg
Cam12/0197 tpleeaikdvdvsgvlryrydtgn--fdknf-v--nnsnlnnskqdhkyraqvnfsaaiadnfkafvqfdynaadg
Cam12/0156 tpleeaikdvdvsgvlryrydtgn--fdknf-v--nnsnlnnskqdhkyraqvnfsaaiadnfkafvqfdynaadg
Cam12/0190 tpleeaikdvdvsgvlryrydtgn--fdknf-v--nnsnlnnnkqdhkyraqvnfsaaiadnfkafiqfdynavdg
Cam12/0202 tpleeaikdvdvsgvlryrydtgn--fdknf-v--nnsnlnnskqdhkyraqvnfsaaiadnfkafvqfdynaadg
Cam12/0222 tpleeaikdvdvsgvlryrydtgn--fdknf-v--nnsnlnnnkqdhkyraqvnfsaaiadnfkafiqfdynavdg
Cam12/0183 tpleeaikdvdvsgvlryrydtgn--fdknf-v--nnsnlnnnkqdhkyraqlnfsaaiadnfkafiqfdynavdg

52/P       tpleeaikdidvsgvlryryetsnewsding-vaqnegsgisgkqdhkyraqlnfsgaiadnfkafvqldynakdg
70/P       tpleeaikdidvsgvlryryetsnewsding-vaqnegsgisgkqdhkyraqlnfsgaiadnfkafvqldynakdg
K43/5      tpleeaikdidvsgvlryryetsnewsding-vaqnegsgisgkqdhkyraqlnfsgaiadnfkafvqldynakdg
KC7        tpleeaikdidvsgvlryryetsnewsding-vaqnegsgisgkqdhkyraqlnfsgaiadnfkafvqldynakdg
MB3361     tpleeaikdidvsgvlryrydtgr--fgkdlnl--enanlnsskqehkyraqinfsgaiadnfkafvqldynakdg
                            β1                L1               β2               β3

KC40       gygtne-------ik-------ndqkglfvrqlyltytnedvatsviagkqqlntiwtdngidglvgtgvkvinns
7P-6.12    gtgvdn-------et-------naekglfvrqlyltytnedvatsviagkqqlnliwtdnaidglvgtgvkvvnns
10C-6.1    gtgvdn-------vk-------naekglfvrqlyltytnedvatsviagkqqlnliwtdnaidglvgtgvkvvnns
10KF-1.16  gygang-------ik-------ndqkglfvrqlyltytnedvatsviagkqqlntiwtdngvdglvgtgikvvnns
10KF-4.12  gygtds-------is-------ntsdtltvrqlyltytnedvatsviagkqqlntiwtdngidglvgtgvkvvnns
10-VTDD8   gtgvdn-------vt-------naekglfvrqlyltytnedvatsviagkqqlnliwtdnaidglvgtgvkvvnns
KC59.1     gygang-------ik-------ndqkglfvrqlyltytnedvatsviagkqqlntiwtdngvdglvgtgikvvnns
KC64.1     gygtds-------is-------ntsdtltvrqlyltytnedvatsviagkqqlntiwtdngvdglvgtgikvvnns
KC67.2     gygang-------ik-------ndqkglfvrqlyltytnedvatsviagkqqlntiwtdngvdglvgtgikvvnns
KC84.2     gygads-------is-------ntsdtlsvrqlyltytnedvatsviagkqqlntiwtdntidglvgtgikvinns
KC96.1     gygang-------ik-------ndqkglfvrqlyltytnedvatsviagkqqlnliwtdnaidglvgtgvkvvnns
KC101      gygang-------ik-------ndqkglfvrqlyltytnedvatsviagkqqlnliwtdnaidglvgtgvkvvnns

Cam12/0214 gygang-------ik-------ndqkglfvrqlyltytnedvatsviagkqqlnliwtdnaidglvgtgvkvvnns
Cam12/0231 gtgvdn-------kt-------naekglfvrqlyltytnedvatsviagkqqlnfiwtdnaidglvgtgvkvvnns
Cam12/0146 gygang-------ik-------ndqkglfvrqlyltytnedvatsviagkqqlnliwtdnaidglvgtgvkvvnns
Cam12/0152 gtgvnn-------vk-------naekglfvrqlyltytnedvatsviagkqqlnliwtdnaidglvgtgvkvvnns
Cam12/0173 gygtds-------is-------ntsdtltvrqlyltytnedvatsviagkqqlntiwtdngidglvgtgvkvvnns
Cam12/0197 gygang-------ik-------ndqkglfvrqlyltytnedvatsviagkqqlnliwtdnaidglvgtgvkvvnns
Cam12/0156 gygang-------ik-------ndqkglfvrqlyltytnedvatsviagkqqlntiwtdngvdglvgtgikvvnns
Cam12/0190 gtgvnn-------vk-------naekglfvrqlyltytnedvatsviagkqqlnliwtdnaidglvgtgvkvvnns
Cam12/0202 gygang-------ik-------ndqkglfvrqlyltytnedvatsviagkqqlnliwtdnaidglvgtgvkvvnns
Cam12/0222 gtgvdn-------vt-------naekglfvrqlyltytnedvatsviagkqqlnliwtdnaidglvgtgikvvnns
Cam12/0183 gtgvdn-------vt-------naekglfvrqlyltytnedvatsviagkqqlnliwtdnaidglvgtgvkvvnns

52/P       gygannsgsttrsyta-------dnsstlnvrqlyltytdenvatsvilgkqqlntiwtdnaidglvgtgvkvvnns
70/P       gygannsgsttrsyaa-------dnlstlnvrqlyltytdenvatsvilgkqqlntiwtdnaidglvgtgvkvvnns
K43/5      gygtnngst----tr-------sdssklnvrqlyltytdenvatsvilgkqqlntiwtdnaidglvgtgvkvvnns
KC7        gygann-------ssttrsytadnsstlnvrqlyltytdenvatsvilgkqqlntiwtdnaidglvgtgvkvvnns
MB3361     gygans-------vs-------ntktslnvrqlyltytnedvatsvilgkqqlntiwtdnaidglvgtgikvvnns
                         L2               β4              β5         L3          β6
```

```
KC40          idgltlaafavdsfmaaeqqgadllghsn--------issdkp---stapfk-------------ldsignlyggaa
7P-6.12       idgltlaafavdsfmaaeqqgaellghsn--------istts----nqarfk-------------vdslgnlygaaa
10C-6.1       idgltlaafaadsfmaaeqqgadllghsn--------tstatp---nqapfk-------------vdsvgnlygaaa
10KF-1.16     idgltlaafavdsfmaaeqqgsdlvgang-------------------safk-------------vdsignlygaaa
10KF-4.12     idgltlaafamdsfneasdttvtitqdnnqkitgvqfnrgnpkgdsdvsga-------------ldwskniygaaa
10-VTDD8      idgltlaafavdsfmaaeqqgaellghsn--------istts----nqapfk-------------vdsvgnlygaaa
KC59.1        idgltlaafavdsfmaeeqqgadllgqst--------isttq----naapfk-------------vdsvgnlygaaa
KC64.1        idgltlaafamdsfneasdttvti--tq--------dsnqki---tgvqfnrgnpkgdgdvsgaldwskniygaaa
KC67.2        idgltlaafavdsfmaaeqqgadllghsn--------tstatp---nqvpfk-------------vdsvgnlygaaa
KC84.2        idgltlaafamdsfneasdttvtitqdn----------sqki---tgvqfnrgnpkgdsdvsgaldwskniygata
KC96.1        idgltlaafavdsfmaaeqqgadllghsn--------istts----nqapfk-------------vdsvgnlygaaa
KC101         idgltlaafavdsfmaaeqqgadllghst--------tsttq----kaapfk-------------vdsvgnlygaaa

Cam12/0214 idgltlaafavdsfmaaeqqgadllghsn--------istt-p---nqapfk-------------vdsvgnlygaaa
Cam12/0231 idgltlaafavdsfmaaeqqgaellgrsn--------istts----nqapfk-------------adsvgnlygaaa
Cam12/0146 idgltlaafavdsfmaaeqqgadllghsn--------istt-p---nqapfk-------------vdsvgnlygaaa
Cam12/0152 idgltlaafaadsfmaaeqqgadllghsn--------istts----nqapfk-------------vdsvgnlygaaa
Cam12/0173 idgltlaafamdsfneasdttvtitqdnnqkitgvqfnrgnpkgdsdvsga-------------ldwskniygaaa
Cam12/0197 idgltlaafavdsfmaaeqqgadllghst--------tsttq----kaapfk-------------vdsvgnlygaaa
Cam12/0156 idgltlaafavdsfmaaeqqgsdlvgang-------------------safk-------------vdsignlygaaa
Cam12/0190 idgltlaafaadsfmaaeqqgadllghsn--------istts----nqapfk-------------vdsvgnlygaaa
Cam12/0202 idgltlaafavdsfmaaeqqgadllghst--------tsttq----kaapfk-------------vdsvgnlygaaa
Cam12/0222 idgltlaafaadsfmaaeqqgadllghsn--------istttp---nqapfk-------------vdsvgnlygaaa
Cam12/0183 idgltlaafavdsfmaaeqqgaellghsn--------iptts----nqapfk-------------vdsvgnlygaaa

52/P          idgltlaafavdsynsdeqqgdlgtvln--------fne------------------------------nlygaaa
70/P          idgltlaafavdsynsdeqqgdlgtvln--------fne------------------------------nlygaaa
K43/5         idgltlaafavdsynsdeqigdlgkvln--------fne------------------------------nlygaaa
KC7           idgltlaafavdsynsdeqqgdlgtvln--------fne------------------------------nlygaaa
MB3361        idgltlaafaidsvntdeqqgdgvffkng--------nltdag---dnspy--------------ldwsgniygaaa
                         β7                                    L4                                         β8

KC40          vgsyeflggqfnpqlwlaywdqvaffyavdaaysttifdginwtlegaylgnsidselddd--k---tht-ngnlfa
7P-6.12       vgsydlaggqfnpqlwlaywdqvaffyavdaaysttifdginwtiegaylgnsidselddd--t---tht-ngnffa
10C-6.1       vgsydlaggqfnpqlwlaywdqvaffyavdaaysttifdginwtlegaylgnsldselddd--t---tha-ngnlfa
10KF-1.16     vgsydlaggqfnpqlwlaywdqvaffyaldasysttifdginwtlegaylgnsldsel-d--k---tya-ngnlfa
10KF-4.12     igsydiaggqfnpqlwlaymsdnaflyaldaaysttifdginwtiegaylgnsvdnklkd--r---ldaangnffa
10-VTDD8      vgsydlaggqfnpqlwlaywdqvaffyavdaaysttifdginwtlegaylgnsldselddd--k---tha-ngnlfa
KC59.1        vgsydlaggqfnpqlwlaywdqvaffyavdaaysttifdginwtlegaylgnsldselddd--k---tha-ngnlfa
KC64.1        igsyditggqfnpqlwlaymsdnaflyaldaaysttifdginwsiegaylgnsvdnklkd--r---lgvangnffa
KC67.2        vgsydlaggqfnpqlwlaywdqvaffyavdaaysttifdginwtlegaylgnsldselddd--t---tha-ngnlfa
KC84.2        igsydiaggqfnpqlwlaymsdnaflyaldatysttifdginwtiegaylgnsvdnklkd--r---ldaangnffa
KC96.1        vgsydlaggqfnpqlwlaywdqvaffyavdaaysttifdginwtlegaylgnsldselddd--k---tha-ngnlfa
KC101         vgsydlaggqfnpqlwlaywdqvaffyavdaaysttifdginwtlegaylgnsldselddd--k---tha-ngnlfa

Cam12/0214 vgsydlaggqfnpqlwlaywdqvaffyavdaaysttifdginwtlegaylgnsldselddd--k---tha-ngnlfa
Cam12/0231 vgsydlaggqfnpqlwlaywdqvaffyavdaaysttifdginwtiegaylgnsidselddd--t---tht-ngnffa
Cam12/0146 vgsydlaggqfnpqlwlaywdqvaffyavdaaysttifdginwtlegaylgnsldselddd--k---tha-ngnlfa
Cam12/0152 vgsydlaggqfnpqlwlaywdqvaffyavdaaysttifdginwtlegaylgnsldselddd--k---tha-ngnlfa
Cam12/0173 igsydiaggqfnpqlwlaymsdnaflyaldaaysttifdginwtiegaylgnsvdnklkd--r---ldaangnffa
Cam12/0197 vgsydlaggqfnpqlwlaywdqvaffyavdaaysttifdginwtlegaylgnsldselddd--k---tha-ngnlfa
Cam12/0156 vgsydlaggqfnpqlwlaywdqvaffyaldasysttifdginwtlegaylgnsldsel-d--k---tya-ngnlfa
Cam12/0190 vgsydlaggqfnpqlwlaywdqvaffyavdaaysttifdginwtlegaylgnsldselddd--k---tha-ngnlfa
Cam12/0202 vgsydlaggqfnpqlwlaywdqvaffyavdaaysttifdginwtlegaylgnsldselddd--k---tha-ngnlfa
Cam12/0222 vgsydlaggqfnpqlwlaywdqvaffyavdaaysttifdginwtlegaylgnsldselddd--k---tha-ngnlfa
Cam12/0183 vgsydlaggqfnpqlwlaywdqvaffyavdaaysttifdginwtlegaylgnsldselddd--k---tha-ngnlfa

52/P          igsyevfnggqlnpqlwlaymtdnaffyavdaayntttifdgvnwtlegaylgnsldnerkd--l---gng-ngnffa
70/P          igsyevfnggqlnpqlwlaymtdnaffyavdaayntttifdgvnwtlegaylgnsldnerkd--l---gng-ngnffa
K43/5         igsyevfnggqlnpqlwlaymtdnaffyavdaayntttifdgvnwtlegaylgnsldnerkd--l---gng-ngnffa
KC7           igsyevfnggqlnpqlwlaymtdnaffyavdaayntttifdgvnwtlegaylgnsldnerkdlgn---gng-ngnffa
MB3361        igsyevfnggqlnpqlwlaymtdnaflyaldvayntttifdgvnwtlegaylgnsldntlddd--yyagrsa-ngnffg
                                    β9               β10                  β11              L5
```

```
KC40         lrgsvelngwdaslgglyygdkekastvviedqgnigsllageeifyttgsrlngdtgrnifgyvtggytfnetvr
7P-6.12      lkggievngwdaslgglyygdkekastvviedqgnlssllageeifyttgsrlngdtgrnifgyvtggytfnetvr
10C-6.1      lkgsievngwdaslgglyygdkekastvviedqgnlgsllageeifyttgsrlngdtgrnifgyvtggytfnetvr
10KF-1.16    lkgsievngwdaslgglyygdkekastvviedqgnigsllageeifyttgsrlngdtgrnifgyvtggytfnetvr
10KF-4.12    lrgtvevngwdaslgglyygkkdkvtlttiedqgnlgsllageeifytngsnlngdigrnifgyvtagytfnetvr
10-VTDD8     lkgsievngwdaslgglyygdkekastvviedqgnlgsllaggeifyttgsrlngdtgrnifgyvtggytfnetvr
KC59.1       lkgsievngwdaslgglyygdkekastvviedqgnlgsllageeifyttgsrlngdtgrnifgyvtggytfnetvr
KC64.1       lrgtvevngwdaslgglyygkkdkvtvttiedqgnlgsllageeifytrgsnlngdlgrnifgyvtggytfneavr
KC67.2       lkgsievngwdaslgglyygdkekastvviedqgnlgsllageeifyttgsrlngdtgrnifgyvtggytfnetvr
KC84.2       lrgtvevngwdaslgglyygkkdkitvttiedqgnlgsllageeifytrgsnlngdlgrnifgyvtggytfneavr
KC96.1       lkgsievngwdaslgglyygdkekastvviedqgnlgsllageeifyttgsrlngdtgrnifgyvtggytfnetvr
KC101        lkgsievngwdaslgglyygdkekastvviedqgnlgsllageeifyttgsrlngdtgrnifgyvtggytfnetvr

Cam12/0214 lkgsievngwdaslgglyygdkekastvviedqgnlgsllageeifyttgsrlngdtgrnifgyvtggytfnetvr
Cam12/0231 lkggievngwdaslgglyygdkekastvviedqgnlgsllageeifyttgsrlngdtgrnifgyvtggytfnetvr
Cam12/0146 lkgsievngwdaslgglyygdkekastvviedqgnlgsllageeifyttgsrlngdtgrnifgyvtggytfnetvr
Cam12/0152 lkgsievngwdaslgglyygdkekastvviedqgnlgsllageeifyttgsrlngdtgrnifgyvtggytfnetvr
Cam12/0173 lrgtvevngwdaslgglyygkkdkvtlttiedqgnlgsllageeifytngsnlngdigrnifgyvtagytfnetvr
Cam12/0197 lkgsievngwdaslgglyygdkekastvviedqgnlgsllageeifyttgsrlngdtgrnifgyvtggytfnetvr
Cam12/0156 lkgsievngwdaslgglyygdkekastvviedqgnigsllageeifyttgsrlngdtgrnifgyvtggytfnetvr
Cam12/0190 lkgsievngwdaslgglyygdkekastvviedqgnlgsllageeifyttgsrlngdtgrnifgyvtggytfnetvr
Cam12/0202 lkgsievngwdaslgglyygdkekastvviedqgnlgsllageeifyttgsrlngdtgrnifgyvtggytfnetvr
Cam12/0222 lkgsievngwdaslgglyygdkekastvviedqgnlgsllageeifyttgsrlngdtgrnifgyvtggytfnetvr
Cam12/0183 lkgsievngwdaslgglyygdkekastvviedqgnlgsllageeifyttgsrlngdtgrnifgyvtggytfnetvr

52/P         lngsievngwdatlgglyygkkdkatvtviedqgnlgsllageeifytngsklngdtgrnifgfvkagytfnetvr
70/P         lngsievngwdatlgglyygkkdkatvtviedqgnlgsllageeifytngsklngdtgrnifgfvkagytfnetvr
K43/5        lngsievngwdatlgglyygkknkatvtviedqgnlgsllageeifytngsklngdtgrnifgfvkagytfnetvr
KC7          lngsievngwdatlgglyygkkdkatvtviedqgnlgsllageeifytngsklngdtgrnifgfvkagytfnetvr
MB3361       lrgsvevngwdatlgglyygkkdkvtvtvledqgnigsllageeifytngsklngdtgrnifgyvkagytfnetvr
              β12        β13                     L6                     β14

KC40         vgadfvyggtktetag-hlgggkkleavarvdykyspklnfsafysyvnld
7P-6.12      vgadfvyggtkteaan-hlgggkkleavarvdykyspklnfsafysyvnla
10C-6.1      vgadfvyggtkteaas-hlgggkkleavarvdykyspklnfsafysyvnld
10KF-1.16    vgadfvyggtkteaan-hlgggkkleavarvdykyspklnfsafysyvnld
10KF-4.12    vgadfvyggtktniig-q--ggkkleavarvdykyspklnfsafysyvnvd
10-VTDD8     vgadfvyggtkteaas-hlgggkkleavarvdykyspklnfsafysyvnld
KC59.1       vgadfvyggtkteaan-hlgggkkleavarvdykyspklnfsafysyvnld
KC64.1       vgadfvyggtktniig-q--ggkkleavarvdykyspklnfsafysyvnvd
KC67.2       vgadfvyggtkteaan-hlgggkkleavarvdykyspklnfsafysyvnld
KC84.2       vgadfvyggtktniig-q--ggkkleavarvdykyspklnfsafysyvnvd
KC96.1       vgadfvyggtkteaan-hlgggkkleavarvdykyspklnfsafysyvnld
KC101        vgadfvyggtkteaan-hlgggkkleavarvdykyspklnfsafysyvnld

Cam12/0214 vgadfvyggtkteaan-hlgggkkleavarvdykyspklnfsafysyvnld
Cam12/0231 vgadfvyggtkteaas-hlgggkkleavarvdykyspklnfsafysyvnld
Cam12/0146 vgadfvyggtkteaan-hlgggkkleavarvdykyspklnfsafysyvnld
Cam12/0152 vgadfvyggtkteaan-hlgggkkleavarvdykyspklnfsafysyvnld
Cam12/0173 vgadfvyggtktniig-q--ggkkleavarvdykyspklnfsafysyvnvd
Cam12/0197 vgadfvyggtkteaan-hlgggkkleavarvdykyspklnfsafysyvnla
Cam12/0156 vgadfvyggtkteaan-hlgggkkleavarvdykyspklnfsafysyvnld
Cam12/0190 vgadfvyggtkteaan-hlgggkkleavarvdykyspklnfsafysyvnld
Cam12/0202 vgadfvyggtkteaan-hlgggkkleavarvdykyspklnfsafysyvnld
Cam12/0222 vgadfvyggtkteaaanhlgggkkleavarvdykyspklnfsafysyvnld
Cam12/0183 vgadfvyggtkteaan-hlgggkkleavarvdykyspklnfsafysyvnld

52/P         vgadfvyggtkte----nhsggdkleavarvdykyspklnfsafysyvnvd
70/P         vgadfvyggtkte----nrsggdkleavarvdykyspklnfsafysyvnvd
K43/5        vgadfvyggtktk----nhsggdkleavarvdykyspklnfsafysyvnvd
KC7          vgadfvyggtkte----nhsggdkleavarvdykyspklnfsafysyvnvd
MB3361       vgadfvyggtktesnt-yvgggdkleavarvsykyspklnfsawysyvnvd
                β15         L7        β16            β17
```
