# Supplementary material for: Nanobodies targeting conserved epitopes on the major outer membrane protein of Campylobacter as potential tools for control of Campylobacter colonization
Source: Vet Res. 2017 Dec 8;48:86. doi: 10.1186/s13567-017-0491-9 (PMC5721652; doi:10.1186/s13567-017-0491-9)
Supplement: Supplementary file 8 — Additional file 8. Sequence variability in L3 and L6 of the MOMP encoding sequences from C. jejuni and C. coli strains. For the analysis of L3, sequences were obtained from the NCBI database using Blastp with the MOMP encoding sequence of C. jejuni KC40 as a query. The analysis of L6, was based on porA sequences in the MLSTdb database. [file 13567_2017_491_MOESM8_ESM.pdf]

Sequence variability in L3 and L6 of MOMP encoding sequences of *C. jejuni* and *C. coli* strains.

Loop 3	
LNTIWTDNGIDGL[1]<br>F AV<br>L D<br>I T<br>S	92.5%<br>(620 of 670)
LNTIWTDNGIDGL[2]<br>MDSL SEDAY<br>GI N YL<br>V V<br>F D<br><br>VGSIWTDDL----<br>LNI NV----<br>VGSIWTDDAI---	7.5%<br>(50 of 670)
Loop 6	
DKEKASTVVIEDQGNIGSLLAGEEIFYTTGSRLNGDTG[3]<br>K D VTLTTL LS Q N N I<br>N N I VNAV G R K L<br>H R ILI K I A<br>F D G N<br>T H Q F<br>L E V<br>S Q<br>G<br>Y<br>N	81.5%<br>(1827 of 2230)
DKEKASTVVIEDQGNIGSLLAGEEIFYTTGSRLNGDTG[4]<br>KEDQVTVTTLDNV SLDH AQ LN KR Q HDSQ<br>NGL FNI KEK S T S D N TRNI<br>A I A V Q F N K SVGL<br>Y L G K Q H YCYV<br>G A G E E S K<br>I R E<br>N A S<br>Q R<br>E<br><br>KKDKLTFNTLEDVGNLD--LAGAEIFYTDGSNLNGDIG<br>N E S K V V V<br>T L<br>E<br><br>DKEKASTVVIEDQGNLGSLLAGEEIFYTTGSRLTGSRLNGDTG<br>K D VTLTT N N N N I<br><br>KKDKATITVIEDQGNLGSLLAGEEIFYTNGSKL-----NGGDTG	18.5%<br>(403 of 2230)

Sequence alignment was performed on the L3 and L6 loops of MOMP sequences obtained in this study, from the MLSTdb database and from NCBI. The L3 and L6 loops of MOMP in strain KC40 was utilized as consensus sequences.

[1]Loop 3 amino acid sequence of the MOMP of *C. jejuni* KC40 and the amino acid substitutions found in the other 27 *Campylobacter* isolates, analysed in this study, and in the sequences of the NCBI database at the same positions.

[2]Loop 3 amino acid sequence of the MOMP of *C. jejuni* KC40 and the amino acid substitutions found in the sequences in the NCBI database, which contain amino acid substitutions on different positions than found in the sequences of the MOMPs of the 28 strains analysed in this study.

[3]Loop 6 amino acid sequence of the MOMP of *C. jejuni* KC40 and the amino acid substitutions found in the other 27 *Campylobacter* isolates, analysed in this study, and in the sequences of the MLSTdb database at the same positions.

[4]Loop 6 amino acid sequence of the MOMP of *C. jejuni* KC40 and the amino acid substitutions found in the sequences in the MLSTdb database, which contain amino acid substitutions on different positions than found in the sequences of the MOMPs of the 28 strains analysed in this study.
